# Adoptive T cell therapy cures mice from active hemophagocytic lymphohistiocytosis (HLH)

**DOI:** 10.15252/emmm.202216085

**Published:** 2022-10-24

**Authors:** Kristoffer Weißert, Sandra Ammann, Tamara Kögl, Viviane Dettmer‐Monaco, Christoph Schell, Toni Cathomen, Stephan Ehl, Peter Aichele

**Affiliations:** ^1^ Institute for Immunodeficiency, Medical Center ‐ University of Freiburg, Faculty of Medicine University of Freiburg Freiburg Germany; ^2^ Center for Chronic Immunodeficiency (CCI), Medical Center ‐ University of Freiburg, Faculty of Medicine University of Freiburg Freiburg Germany; ^3^ Faculty of Biology Albert‐Ludwigs‐University of Freiburg Freiburg Germany; ^4^ Institute for Immunology, Medical Center ‐ University of Freiburg, Faculty of Medicine University of Freiburg Freiburg Germany; ^5^ Institute for Transfusion Medicine and Gene Therapy, Medical Center University of Freiburg Freiburg Germany; ^6^ Institute of Surgical Pathology, Medical Center University of Freiburg Freiburg Germany; ^7^ Center for Integrative Biological Signalling Studies Albert‐Ludwigs‐University of Freiburg Freiburg Germany

**Keywords:** adoptive T cell therapy, hemophagocytic lymphohistiocytosis, hyperinflammation, virus‐specific T cells, Haematology, Immunology

## Abstract

Primary hemophagocytic lymphohistiocytosis (HLH) is a hyperinflammatory syndrome caused by impaired lymphocyte cytotoxicity. First‐line therapeutic regimens directed against activated immune cells or secreted cytokines show limited efficacy since they do not target the underlying immunological problem: defective lymphocyte cytotoxicity causing prolonged immune stimulation. A potential rescue strategy would be the adoptive transfer of *ex vivo* gene‐corrected autologous T cells. However, transfusion of cytotoxicity‐competent T cells under conditions of hyperinflammation may cause more harm than benefit. As a proof‐of‐concept for adoptive T cell therapy (ATCT) under hyperinflammatory conditions, we transferred syngeneic, cytotoxicity‐competent T cells into mice with virally triggered active primary HLH. ATCT with functional syngeneic trigger‐specific T cells cured *Jinx* mice from active HLH without life‐threatening side effects and protected *Perforin*‐deficient mice from lethal HLH progression by reconstituting cytotoxicity. Cured mice were protected long‐term from HLH relapses. A threshold frequency of transferred T cells with functional differentiation was identified as a predictive biomarker for long‐term survival. This study is the first proof‐of‐concept for ATCT in active HLH.

## Graphical abstract

Primary hemophagocytic lymphohistiocytosis (HLH) is a life‐threatening syndrome characterized by hyperinflammation and caused by impaired lymphocyte cytotoxicity. We established adoptive immunotherapy in HLH animal models under conditions of hyperinflammation.

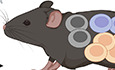

**The paper explained**

**Problem**

Deficiency in lymphocyte cytotoxicity predisposes to the development of a hyperinflammatory syndrome: primary hemophagocytic lymphohistiocytosis (HLH). First‐line therapy of HLH patients focusses on strong immunosuppression to control hyperinflammation by eliminating hyperactivated immune cells and dampening the cytokine storm. However, these therapeutic interventions often show limited efficacy since they do not correct the underlying immunological defect. Thus, the survival of HLH patients is not satisfactory due to uncontrolled disease activity, treatment toxicity, and relapses. Here, we evaluated a new therapeutic strategy based on the transfer of functional T cells to partially reconstitute the cytolytic function in HLH‐prone mice with active HLH.

**Results**

Functional virus‐specific T cells were transferred into mice with active virus‐triggered HLH. The transferred T cells cured *Jinx* mice from hyperinflammation and protected *Perforin*‐deficient mice from lethal HLH progression by eliminating the HLH‐inducing viral trigger and silencing the disease process without life‐threatening side effects. Cured mice were protected from HLH relapses long‐term. A distinct differentiation status of transferred T cells was identified to predict therapeutic success already 10 days after therapy start.

**Impact**

Although it seems counterintuitive to enhance immunity under conditions of hyperinflammation, this study is the first proof‐of‐concept for successful curative T cell therapy in mice with active HLH. These promising results in the animal models are a prerequisite for future innovative clinical protocols based on the transfer of *ex vivo* gene‐corrected autologous T cells in HLH patients.

## Introduction

Hemophagocytic lymphohistiocytosis (HLH) is a life‐threatening immunohematologic disorder (estimated incidence 1:50,000–1:100,000) (Henter *et al*, 1991; Meeths *et al*, 2015) caused by hyperactivated T cells and macrophages, leading to excessive inflammation and multi‐organ failure. In primary HLH, uncontrolled immune activation is the result of gene defects affecting perforin‐dependent cytotoxicity of NK and CD8 T cells (Schmid *et al*, 2010; Janka & Lehmberg, 2014; de Saint Basile *et al*, 2015; Chinn *et al*, 2018). As a consequence, the lysis of APCs is impaired, leading to continuous T cell stimulation, excessive cytokine release, macrophage hyperactivation, and immunopathology. HLH patients present with prolonged fever, hepatosplenomegaly, cytopenia, often liver disease and neurological manifestations (Bergsten *et al*, 2020; Wegehaupt *et al*, 2020). Most FHL patients are born asymptomatically and activate during the first year of life, likely in the context of a trigger. In around 20% of primary HLH cases, this is an infectious trigger, with EBV and CMV being the main culprits (Heeg *et al*, 2018). In the other 80%, no infectious trigger is identified at the time of diagnosis. It is currently unclear whether infectious or inflammatory agents escape detection or have already been eliminated at the time of diagnosis. If untreated, the outcome of primary HLH is generally fatal (Janka, 1983; Canna & Marsh, 2020; Meeths & Bryceson, 2021). Clinical management of HLH patients is divided into two main phases. Phase I (first‐line therapy) focusses on remission, control of trigger, maintenance of remission, and salvage therapies in case of relapsing or refractory HLH. After initial remission is achieved, phase II aims at curing the underlying genetic defect by allogeneic hematopoietic stem cell transplantation (HSCT). First‐line therapy uses initial aggressive immunosuppression based on etoposide (HLH‐94 protocol) or antithymocyte globulin (ATG) (Henter *et al*, 2007; Mahlaoui *et al*, 2007; Ehl *et al*, 2018). Promising results have also been achieved with the monoclonal anti‐CD52 antibody (alemtuzumab; Moshous *et al*, 2019). These remission‐inducing therapies suppress hyperinflammation, eliminate activated immune cells and dampen the cytokine storm, stabilizing the patients for HSCT. Novel therapeutic approaches targeting key disease‐driving cytokines like IFNγ (emapalumab) (Jordan *et al*, 2004; Vallurupalli & Berliner, 2019; Locatelli *et al*, 2020) or cytokine receptor signaling pathways like JAK1/2 (ruxolitinib) (Das *et al*, 2016; Maschalidi *et al*, 2016; Z*hang et al*, 2020; Keenan *et al*, 2021) frequently require rescue therapies when used as single agents for primary HLH. Thus, the overall survival of HLH patients is not satisfactory (about 60%), due to uncontrolled disease activity, treatment toxicity, relapses before transplantation, and opportunistic infections (Bergsten *et al*, 2017; Ehl, 2017).

Hence, innovative therapeutic strategies are urgently needed to improve phase I of HLH therapy for sustained control of hyperinflammation. So far, all remission‐inducing therapies preparing for HSCT, do not address the critical underlying immunological problem: defective lymphocyte cytotoxicity causing continuous immune stimulation due to prolonged antigen presentation. Partial reconstitution of cytolytic activity by transfer of functional T cells (adoptive T cell therapy, ATCT) to reconstitute the negative feedback loop by eliminating stimulating cells and terminating continuous triggering may stop the vicious cycle. In principle, this could be achieved by harvesting gene‐deficient T cells from individuals undergoing HLH in order to get autologous (syngeneic), trigger‐antigen‐experienced T cells, to correct the genetic defect by gene addition or gene editing and then to re‐infuse the T cells (Panchal *et al*, 2021). Infusion of autologous (syngeneic) antigen‐experienced T cells is a therapeutic strategy to fight cancer, infectious diseases, and genetic immune disorders (Weber *et al*, 2020). Such innovative clinical protocols are under development for XLP, FHL‐2, and FHL‐3 patients (Soheili *et al*, 2016; Tiwari *et al*, 2016; Ghosh *et al*, 2018; Panchal *et al*, 2018; Takushi *et al*, 2020). However, there are major concerns about fuelling the hyperinflammation by adding cytokine‐producing immune effector cells.

HLH‐prone mice are excellent models for primary HLH and have been absolutely essential to understand the pathogenesis of this disease (Jordan *et al*, 2004; Jessen *et al*, 2011, 2013; Kögl *et al*, 2013; Terrell & Jordan, 2013a). Mice do not develop disease spontaneously, but infections with lymphocytic choriomeningitis virus (LCMV) or murine cytomegalovirus (MCMV), both systemic persistent viruses like EBV and CMV in patients, trigger HLH with all criteria used to define the human disease (Jordan *et al*, 2004; Jessen *et al*, 2011; Gather *et al*, 2020). MUNC13‐4‐deficient *Jinx* mice are a preclinical model for familial HLH type 3 (FHL‐3). They harbor a point mutation in the *Unc13d* gene coding for a dysfunctional MUNC13‐4 protein leading to impaired cytotoxicity (Crozat *et al*, 2007). *Perforin*‐deficient (PKO) mice are a preclinical model for FHL‐2 and are defective in perforin, which creates pores in the target cell membrane to facilitate apoptotic cell death (Kägi *et al*, 1994a, 1994b; Jordan *et al*, 2004).

Here we used these two preclinical HLH mouse models to test the efficacy and safety of trigger‐specific ATCT to reconstitute immunity under conditions of hyperinflammation during active HLH. This is an important advance over previous work using prophylactic adoptive transfer of polyclonal WT or gene‐corrected CD8 T cells into HLH‐prone mice before disease induction to protect from HLH (Terrell & Jordan, 2013b; Ghosh *et al*, 2018). ATCT with CD3 T cells isolated from LCMV‐immune WT mice cured *Jinx* mice from the clinical symptoms of active HLH without inducing life‐threatening side effects, while establishing substantial long‐term donor CD8 T cell chimerism. Mice with ATCT were protected from HLH relapses, even when re‐exposed to LCMV infection as a disease trigger. A threshold frequency of T cells with “functional” differentiation was defined as a predictive biomarker for therapeutic success in mice with active HLH already 10 days after therapy start. Results were confirmed in PKO mice rescuing them from acute and lethal HLH progression. Thus, our study provides proof‐of‐concept that ATCT may serve as a remission‐inducing therapy for primary HLH.

## Results

### *Jinx* mice develop acute HLH turning into chronic disease with late lethality

**Figure 1:**
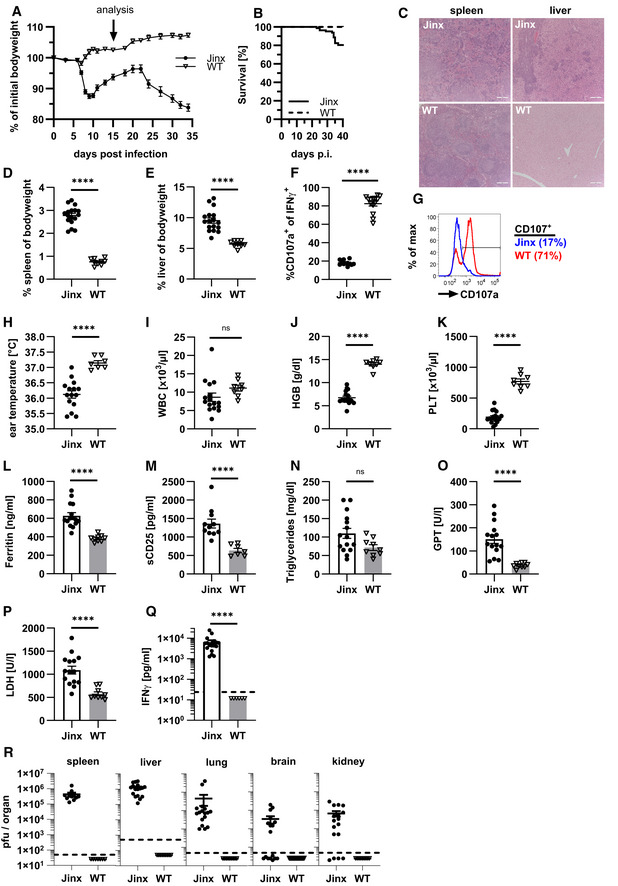
Nonfatal active HLH in *Jinx* mice on day 15 post infection *Jinx* mice and heterozygous littermates (WT) were infected with 200 pfu LCMV‐WE intravenously (i.v.).
A, BBodyweight (A) and survival (B) of mice were monitored for 5 weeks (*n* (A) = 43 *Jinx*, 52 WT; *n* (B) = 88 *Jinx*, 73 WT).CSpleen and liver sections (day 15–21 post infection (p.i.)) were stained with *hematoxylin and eosin* (HE) (*n* = 6–7 mice per group).D, ESpleen and liver weight in relation to bodyweight were analyzed on day 15 p.i. (*n* = 17 *Jinx*, 9 WT).F, GDegranulation capacity of CD8 T cells on day 8 p.i. after restimulation with LCMV‐GP_33–41_: Frequency of CD107a^+^ of all IFNγ^+^ CD8 T cells. The following analyses were performed on day 15 p.i. (*n* = 12 *Jinx*, 14 WT).H–KEar temperature, frequency of white blood cells (WBC), concentration of hemoglobin (HGB), and frequency of platelets (PLT) in blood (*n* = 15–17 *Jinx*, 7–8 WT).L–QSerum concentration of ferritin, soluble CD25, triglycerides, glutamate‐pyruvate transaminase (GPT), lactate dehydrogenase (LDH) and interferon γ (IFNγ) (*n* = 15–17 *Jinx*, 6–9 WT).RVirus titres of spleen, liver, lung, brain, and kidney (*n* = 15 *Jinx*, 9 WT). Data information: Horizontal lines in graphs represent mean values. Horizontal dashed line (Q, R) indicates the detection limit. Scale bars are 200 μm long. Data are mean ± SEM with *n* = 6–17 per group in 2–4 independent experiments. Statistics: unpaired *t*‐test (D, E, H, K, N, O), log‐rank test (B), Mann–Whitney test (B, F, I, J, L, M, P, Q), ns (not significant) *P* > 0.05; ****P ≤ 0.0001.

*Jinx* mice develop FHL‐3‐like disease after LCMV infection (Crozat *et al*, 2007). However, a complete evaluation of diagnostic HLH criteria and long‐term disease progression was not reported so far. To define a suitable time point for ATCT, we monitored bodyweight and survival for 5 weeks after infection. A dramatic weight loss was observed starting from day 6 in *Jinx* mice. After a crisis between day 8–12 mice stabilized, followed by the second phase of bodyweight loss and increased mortality. About 20% of *Jinx* mice were eliminated until day 35 according to animal care guidelines, whereas WT littermates survived (Fig 1A and B). Analysis on day 15 revealed a pronounced hepatosplenomegaly in *Jinx* mice (Fig 1D and E) with strong infiltrations of lymphocytes and massive tissue destruction (Fig 1C). Due to defective MUNC13‐4, *Jinx* CD8 T cells showed impaired degranulation capacity as indicated by reduced CD107a surface expression (Fig 1F and G). Fever diagnosed in HLH patients corresponds to a significant decrease in peripheral ear temperature in *Jinx* mice indicating a centralization of circulation. A significant reduction in hemoglobin and platelets was observed in *Jinx* mice (Fig 1H–K). Serum analysis revealed increased ferritin and sCD25, normal triglycerides and elevated liver enzymes (GPT, LDH), as well as high IFNγ levels (Fig 1L–Q). LCMV persisted in *Jinx* mice, in contrast to WT littermates (Fig 1R). In summary, on day 15 after the viral trigger, *Jinx* mice showed the complete clinical picture of active HLH. In this early phase, HLH progression in *Jinx* mice was not fatal in contrast to PKO mice (Jordan *et al*, 2004; Kögl *et al*, 2013).

### CD8 T cell phenotype in HLH mouse models and FHL patients

**Figure EV1:**
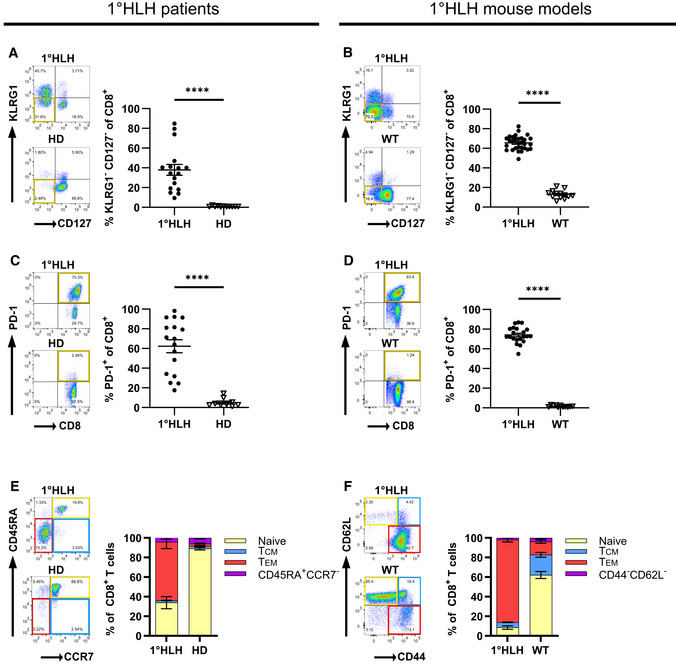
CD8 T cell phenotype in HLH mouse models and FHL patients Left column: CD8 T cells obtained from 1°HLH patients (1°HLH) were analyzed by flow cytometry in comparison to healthy donors (HD). Right column: *Jinx* and PKO mice (1°HLH) were infected with 200 pfu LCMV‐WE intravenously and analyzed by flow cytometry on day 12–15 p.i. in comparison to noninfected wild‐type mice (WT).
A, BThe frequency of CD8 T cells double negative for KLRG1 and CD127 (KLRG1^−^CD127^−^) was determined (A) in blood of 1°HLH patients, HD and (B) in the spleen of 1°HLH mouse models, as well as noninfected WT mice (*n* = 17 1°HLH patients, *n* = 11 HD, *n* = 30 1°HLH mice, *n* = 13 WT mice).C, DFrequency of CD8 T cells expressing PD‐1 was determined (C) in blood of 1°HLH patients in comparison to HD (D) and in the spleen of 1°HLH mouse models, as well as noninfected WT mice (*n* = 17 1°HLH patients, *n* = 11 HD, *n* = 21 1°HLH mice, *n* = 11 WT mice).EExpression of CD45RA and CCR7 on CD8 T cells was determined in blood of 1°HLH patients and HD and four populations were distinguished: CD45RA^+^CCR7^+^ termed “naïve”, CD45RA^−^CCR7^+^ termed “T_CM_”, CD45RA^−^CCR7^−^ termed “T_EM_” and CD45RA^+^CCR7^−^ (*n* = 17 1°HLH patients, *n* = 11 HD).FExpression of CD62L and CD44 on CD8 T cells was determined in the spleen of 1°HLH mouse models, and noninfected WT mice and four populations were distinguished: CD44^−^CD62L^+^ termed “naïve”, CD44^+^CD62L^+^ termed “T_CM_”, CD44^+^CD62L^−^ termed “T_EM_” and CD44^−^CD62L^−^ (*n* = 19 1°HLH mice, *n* = 9 WT mice). Data information: Horizontal lines in graphs represent mean values. Data are mean ± SEM. Statistics: unpaired *t*‐test (B, D), Mann–Whitney test (A, C). ****P ≤ 0.0001. Source data are available online for this figure.

Next, we evaluated whether the phenotype of the disease‐driving CD8 T cells was comparable in patients and the preclinical mouse models. Based on a 1°HLH patient cohort published by Ammann *et al* (2017) we re‐analyzed eight FHL‐2 patients, seven FHL‐3 patients, and two FHL‐5 patients from that cohort. All patients fulfilled at least five out of eight clinical HLH parameters, were less than 1 year old, and were either untreated or close to therapy start (Table 1). For the preclinical mouse models, we collected data from PKO (FHL‐2) and *Jinx* (FHL‐3) mice on day 12 or 15 post infection, respectively. Healthy donors (HD) and noninfected mice were included as controls. According to the expression of KLRG1 and CD127, we detected activated CD8 T cells that strongly downregulated CD127 (IL‐7Rα chain) and KLRG1 in the context of HLH. This double negative (KLRG1^−^/CD127^−^) CD8 T cell population was prominent in patients and mice with acute HLH but not in HD and noninfected mice (Fig EV1A and B). A high frequency of CD8 T cells in patients and mice expressed the inhibitory receptor PD‐1, which was not observed in T cells of HD and noninfected mice (Fig EV1C and D). Furthermore, the majority of CD8 T cells showed an effector memory phenotype (T_EM_) in 1°HLH patients (CD45RA^−^/CCR7^−^) and mice (CD44^+^/CD62L^−^; Fig EV1E and F). Thus, the phenotype of activated T cells observed in preclinical mouse models resembles those of patients with untreated HLH.

**Table 1:** Primary HLH patient cohort.

1°HLH < 1 year with active HLH		
1°HLH	Patient	Age (months)
FHL‐2	1	1
FHL‐2	2	2
FHL‐2	3	2.5
FHL‐2	4	2
FHL‐2	5	1
FHL‐2	6	1
FHL‐2	7	3
FHL‐2	8	1
FHL‐3	9	4
FHL‐3	10	3
FHL‐3	11	2
FHL‐3	12	6.5
FHL‐3	13	1
FHL‐3	14	8
FHL‐3	15	1
FHL‐5	16	1
FHL‐5	17	3

**Figure 2:**
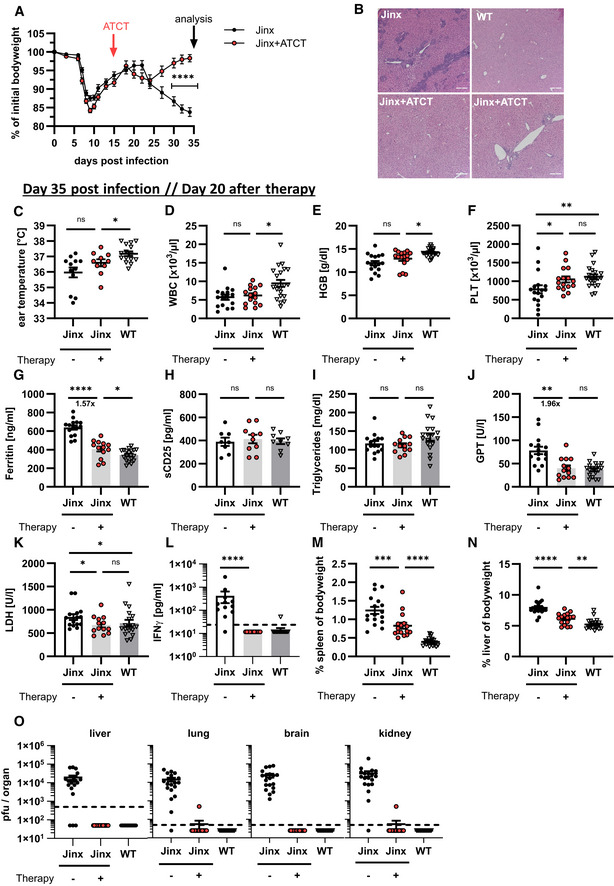
Adoptive T cell therapy cures *Jinx* mice from active HLH *Jinx* mice and heterozygous littermates (WT) were infected with 200 pfu LCMV‐WE i.v. On day 15 p.i., 4 × 10^6^ purified CD3 T cells from LCMV‐immune WT mice were transferred to *Jinx* mice (*Jinx* + ATCT).
ABodyweight was monitored for 5 weeks p.i. (*n* = 42–52 per group).B, CAnalyses performed on day 35 p.i.: (B) Liver sections stained with *hematoxylin and eosin* (HE) (*n* = 3–5 in 1 experiment) and (C) ear temperature (*n* = 12 *Jinx*, 10 *Jinx* + ATCT, 15 WT).D–FFrequency of white blood cells (WBC), concentration of hemoglobin (HGB), and frequency of platelets (PLT) in blood (*n* = 17 *Jinx*, 16 *Jinx* + ATCT, 21 WT).G–LSerum concentration of ferritin, soluble CD25, triglycerides, GPT, LDH, and IFNγ (*n* = 8–15 *Jinx*, 10–13 *Jinx* + ATCT, 9–15 WT).M, NSpleen and liver weight in relation to bodyweight (*n* = 17 *Jinx*, 16 *Jinx* + ATCT, 22 WT).OVirus titres in liver, lung, brain, and kidney (*n* = 19 *Jinx*, 16 *Jinx* + ATCT, 24 WT). Data information: Horizontal lines in graphs represent mean values. Horizontal dashed line (L, O) indicates the detection limit. Scale bars are 200 μm long. Data are mean ± SEM with *n* = 8–24 per group in at least three independent experiments. Statistics: unpaired *t*‐test (A, G, H, J), Mann–Whitney test (C, D, E, F, I, K, L, M, N), ns *P* > 0.05; *P ≤ 0.05; **P ≤ 0.01; ***P ≤ 0.001; ****P ≤ 0.0001.

### ATCT under conditions of hyperinflammation cures *Jinx* mice from active HLH

**Figure EV2:**
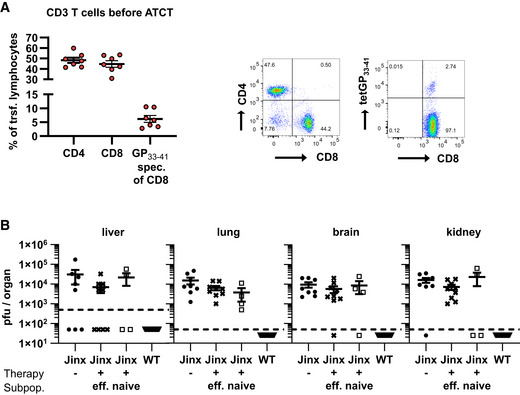
Adoptive T cell therapy with naïve or LCMV‐effector T cells in active HLH is not successful Purified CD3 T cells used for ATCT were analyzed regarding the frequency of CD4 and CD8 T cells and the frequency of LCMV‐GP_33–41_‐specific CD8 T cells among CD8 T cells. Individual dots represent separate transfers/experiments (*n* = 7).
*Jinx* mice (*Jinx*) and heterozygous littermates (WT) were infected with 200 pfu LCMV‐WE intravenously. On day 5–15 p.i., mice remained untreated (*Jinx*, *n* = 8; WT, *n* = 10) or 1 × 10^7^ purified CD3 T cells/lymphocytes were transferred to *Jinx* mice (*Jinx* + eff, *n* = 9). Alternatively, *Jinx* mice received on day 15 p.i. a transfer with 1 × 10^7^ CD3 T cells from uninfected wild‐type mice (*Jinx* + naïve, *n* = 4). Data information: Horizontal lines in graphs represent mean values. Data are mean ± SEM with *n* = 4–10 mice in 1–3 experiments.

This acute HLH with protracted lethality offered an excellent opportunity to evaluate ATCT in *Jinx* mice with the aim to partially reconstitute cytotoxic T cell function. To model the clinical situation of patients, therapy was started under conditions of hyperinflammation during active HLH. Based on the experience that CD8 T cells are dependent on CD4 T cells to help with the resolution of chronic LCMV infections (Matloubian *et al*, 1994; Kalams & Walker, 1998; Aubert *et al*, 2011), we transferred CD3^+^ T cells from LCMV‐immune WT mice (> day 100 after infection) containing virus‐specific memory T cells (Fig EV2A) into diseased *Jinx* mice on day 15 and followed HLH progression. *Jinx* mice with ATCT stabilized and started to gain bodyweight 10 days after therapy start, reaching their initial weight within 20 days. Untreated *Jinx* mice exhibited chronic HLH and bodyweight loss of 15–20% on day 35 p.i. (Fig 2A). *Jinx* mice were analyzed for HLH criteria on day 20 after therapy start. Lymphocyte infiltrations in the liver were strongly reduced in *Jinx* mice after ATCT with only a few infiltrates around the large vessels and restored tissue architecture (Fig 2B). *Jinx* mice without therapy suffered from chronic disease with persistent but incomplete diagnostic HLH criteria, which were originally developed to diagnose acute disease (Fig 2C–N). Platelets, serum ferritin, GPT, LDH, and IFNγ levels had significantly improved in *Jinx* mice undergoing ATCT (Fig 2F, G, J, K, and L). Hepatosplenomegaly declined significantly but did not reach levels of WT controls (Fig 2M and N). The disease process was silenced by reconstituting the negative feedback loop on LCMV‐infected cells, leading to virus elimination and termination of chronic antigen stimulation. The improved clinical status of *Jinx* mice with ATCT correlated with the elimination of the disease trigger LCMV (15/16 mice free of virus; Fig 2O). Of note, ATCT with naïve T cells or effector T cells, obtained from WT mice 5–15 days after LCMV infection, had no curative effect, and as a consequence *Jinx* mice remained persistently infected (Fig EV2B). This indicates that the differentiation state of the donor T cells is relevant for successful curative ATCT in active HLH.

**Figure 3:**
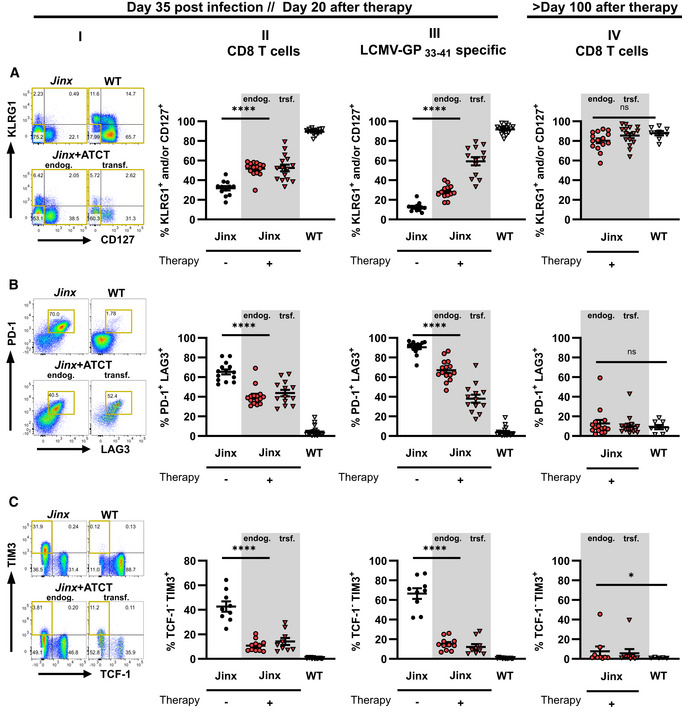
T cell differentiation after ATCT in active HLH *Jinx* mice and heterozygous littermates (WT) were infected with 200 pfu LCMV‐WE i.v. On day 15 p.i., 4 × 10^6^ purified CD3 T cells from LCMV‐immune WT mice were transferred to *Jinx* mice (*Jinx* + ATCT). Transferred CD8 T cells (trsf.) were distinguished from endogenous CD8 T cells (endog.). As controls, *Jinx* and WT mice were left untreated.
A–COn day 35 p.i., endogenous and transferred CD8 T cells (columns I and II), as well as LCMV‐GP_33–41_‐specific CD8 T cells (column III) in the spleen, were analyzed by flow cytometry: (A) frequency of KLRG1^+^ and/or CD127^+^, (B) PD‐1^+^/LAG3^+^ and (C) TCF‐1^−^/TIM3^+^ CD8 T cells (C) The same analyses were performed more than 100 days after therapy/115 days after infection (column IV). Data information: FACS plots are representative of the respective mouse groups. Horizontal lines in graphs represent mean values. Data are mean ± SEM with *n* = 8–15 per group in 2–5 independent experiments. Detailed information *n*: A. (II) *n* = 12 *Jinx*, 15 *Jinx* + ATCT (15× trsf cells), 15 WT in 5 experiments; (III) *n* = 12 *Jinx*, 13 *Jinx* + ATCT (13× trsf cells), 15 WT in 5 experiments; (IV) *n* = 15 *Jinx* + ATCT (15× trsf cells), 8 WT in 3 experiments. B. (II) *n* = 14 *Jinx*, 15 *Jinx* + ATCT (13× trsf cells), 18 WT in 5 experiments; (III) *n* = 14 *Jinx*, 15 *Jinx* + ATCT (13× trsf cells), 18 WT in 3 experiments; (IV) *n* = 15 *Jinx* + ATCT (15× trsf cells), 8 WT in 3 experiments. C. (II) *n* = 9 *Jinx*, 11 *Jinx* + ATCT (9× trsf cells), 13 WT in 4 experiments; (III) *n* = 9 *Jinx*, 11 *Jinx* + ATCT (9× trsf cells), 13 WT in 4 experiments; (IV) *n* = 9 *Jinx* + ATCT (9× trsf cells), 5 WT in 2 experiments. Statistics: unpaired *t*‐test (A column IV, B column II, C column II and III), Mann–Whitney test (A column II, III, B column III and IV, C column IV), ns *P* > 0.05; *P ≤ 0.05; ****P ≤ 0.0001.

### CD8 T cell phenotype in *Jinx* mice with ATCT

Cytotoxicity‐deficient CD8 T cells are the main driver of LCMV‐triggered primary HLH in mice. We analyzed the differentiation of total splenic CD8 T cells in *Jinx* mice with and without ATCT on day 20 (Fig 3, columns I and II) and on day > 100 (Fig 3, column IV) after therapy. In parallel, LCMV‐GP_33–41_‐specific CD8 T cells were analyzed to focus on cells with specificity for the antigenic trigger (Fig 3, column III). Transferred CD8 T cells (trsf.) were distinguished from endogenous T cells (endog.) by congenic markers CD45.1/2 or Thy1.1/2.

Effector T cells were classified based on KLRG1 and CD127 expression (Voehringer *et al*, 2001; Thimme *et al*, 2005; Joshi *et al*, 2007; Sarkar *et al*, 2008). In contrast to WT mice with early infection control, KLRG1^+^ and/or CD127^+^ CD8 T cells were drastically reduced in *Jinx* mice without ATCT. *Jinx* mice undergoing ATCT exhibited significantly increased frequencies of transferred and endogenous T cells with this phenotype (Fig 3A, columns I and II). Analysis of LCMV‐GP_33–41_‐specific T cells demonstrated a high frequency of therapeutic T cells in a KLRG1^+^ and/or CD127^+^ state and a slight re‐differentiation of endogenous T cells compared with T cells in *Jinx* mice without ATCT (Fig 3A, column III). We therefore hypothesized, that KLRG1 and/or CD127 expression were associated with a “functional” antiviral effector phenotype, while loss of expression indicated poor functionality.

**Figure EV3:**
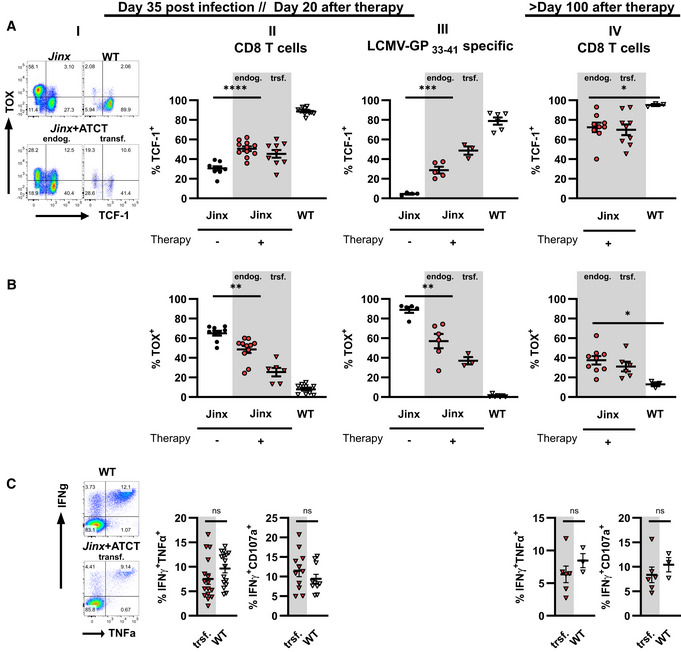
Expression of transcription factors and functionality of T cells after adoptive T cell therapy in active HLH *Jinx* mice (*Jinx*) and heterozygous littermates (WT) were infected with 200 pfu LCMV‐WE intravenously. On day 15 post infection (p.i.), mice remained untreated (*Jinx*, WT) or 4 × 10^6^ purified CD3 T cells from LCMV‐immune WT mice were transferred to *Jinx* mice (Jinx + ATCT). Transferred CD8 T cells (trsf.) were distinguished from endogenous CD8 T cells (endog.).
A, BOn day 20 after therapy, endogenous and transferred CD8 T cells (column II), as well as LCMV‐GP_33–41_‐specific CD8 T cells (column III), were analyzed by flow cytometry: frequency of TCF‐1^+^ or TOX^+^. The same analyses were performed more than 100 days after therapy (column IV).CSplenocytes were restimulated with LCMV‐GP_33–41_. The frequencies of transferred WT CD8 T cells in *Jinx* recipients and WT CD8 T cells expressing IFNγ and TNFα or IFNγ and CD107a after restimulation were determined on day 20 or > 100 days after therapy start (C, columns II, IV). Data information: Horizontal lines in graphs represent mean values. ns *P* > 0.05; *P ≤ 0.05; **P ≤ 0.01; ***P ≤ 0.001; ****P ≤ 0.0001. Data are mean ± SEM with *n* (A–C) 3–19 mice in 1–5 experiments. Statistics: unpaired *t*‐test (A column II, III, IV; B column IV), Mann–Whitney test (B column II, III; C column II, IV). Detailed information *n*: A. (II) *n* = 9 *Jinx*, 11 *Jinx* + ATCT (9× trsf. cells), 11 WT in 4 experiments; (III) *n* = 4 *Jinx*, 5 *Jinx* + ATCT (3× trsf. cells), 6 WT in 2 experiments; (IV) *n* = 10 *Jinx* + ATCT, 3 WT in 2 experiments. B. (II) *n* = 9 *Jinx*, 11 *Jinx* + ATCT (6× trsf. cells), 11 WT in 4 experiments; (III) *n* = 5 *Jinx*, 6 *Jinx* + ATCT (3× trsf. cells), 7 WT in 2 experiments; (IV) *n* = 9 *Jinx* + ATCT (6× trsf. cells), 3 WT in 2 experiments. C. (II) IFNγ/TNFα: *n* = 16 trsf. cells in *Jinx* + ATCT, 16 WT in 5 experiments; (II) IFNγ/CD107a: *n* = 11 trsf. cells in *Jinx* + ATCT, 10 WT in 3 experiments; (II) IFNγ/TNFα: *n* = 6 trsf. cells in *Jinx* + ATCT, 3 WT in 1 experiment; (IV) IFNγ/CD107a: *n* = 6 trsf. cells in *Jinx* + ATCT, 3 WT in 1 experiment.

To further corroborate this finding, we analyzed inhibitory receptors PD‐1 and LAG3, which are associated with stepwise downregulation of T cell functions (Blank *et al*, 2019). In *Jinx* mice undergoing ATCT, the frequencies of transferred and endogenous CD8 T cells expressing PD‐1/LAG‐3 were significantly reduced compared with CD8 T cells in *Jinx* mice without therapy (Fig 3B, columns I and II). Importantly, the frequency of transferred GP33‐specific CD8 T cells expressing PD‐1/LAG3 was considerably lower compared with the endogenous T cells (Fig 3B, column III), indicating a higher “functional” state of the therapeutic T cell population. In line with this finding, “terminally/transitory” exhausted T cells characterized by low TCF‐1 and high TIM3 expression (TCF‐1^−^/TIM3^+^; Im & Ha, 2020) were strongly reduced in transferred and endogenous T cells in *Jinx* mice undergoing ATCT (Fig 3C, column I–III). Accordingly, the frequency of “stem‐like” TCF‐1^+^ CD8 T cells, that exhibit self‐renewal capacity, was increased especially in the transferred therapeutically active GP33‐specific T cells, whereas the frequency of TOX‐expressing T cells was significantly reduced (Fig EV3A and B, columns I–III). On day 20 or > 100 days after therapy start, the transferred CD8 T cells in *Jinx* mice and the CD8 T cells in WT mice were comparable in their capacity to secrete effector cytokines (IFNγ/TNFα) and to degranulate (CD107a; Fig EV3C). Of note, the endogenous, cytotoxicity‐defective CD8 T cells eventually re‐differentiated and aligned to the phenotype of transferred T cells. As a consequence, > 100 days after therapy start, no significant differences in the differentiation phenotype of transferred and endogenous T cells in cured *Jinx* mice versus T cells in WT mice were detectable (Fig 3A–C, column IV). However, the percentage of CD8 T cells expressing the transcription factors TCF‐1 or TOX in cured *Jinx* mice did not normalize to the percentages in WT mice (Fig EV3A and B, column IV). Taken together, our results show, that the therapeutic CD8 T cells retained a functional state with self‐renewal capacity, which is a prerequisite to cure *Jinx* mice from active HLH under conditions of hyperinflammation. During therapy, a certain re‐differentiation of the endogenous CD8 T cells was observed with a partial downregulation of inhibitory receptors and a significantly lower frequency of “terminally/transitory” exhausted T cells (TCF‐1^−^/TIM3^+^), due to the elimination of the continuous trigger by reconstituting the negative feedback loop on virus‐infected cells. As a consequence, the endogenous T cells were silenced and did no longer fuel the disease process.

**Figure 4:**
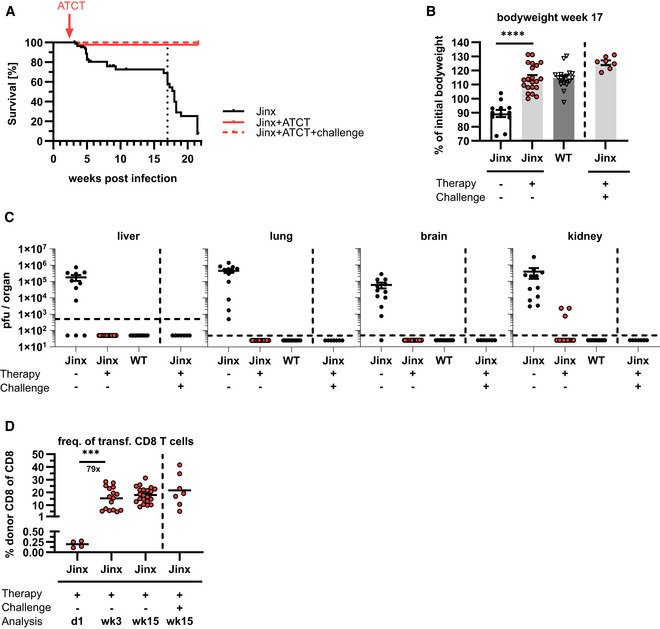
Adoptive T cell therapy cures *Jinx* mice from HLH and protects against HLH relapses *Jinx* mice and heterozygous littermates (WT) were infected with 200 pfu LCMV‐WE i.v. On day 15 p.i. 4 × 10^6^ CD3 T cells from LCMV‐immune WT mice were transferred to *Jinx* mice (*Jinx* + ATCT). As controls, *Jinx* and WT mice were left untreated. A further experimental group was re‐challenged with 10^5^–10^6^ pfu LCMV‐Armstrong intraperitoneally (*Jinx* + ATCT+challenge) more than 45 days after therapy.
AMouse survival was followed for 22 weeks p.i. or postchallenge (*n* = 88 *Jinx*, 43 *Jinx* + ATCT, 7 *Jinx* + ATCT + challenge; *n* (survival until week 22 p.i./challenge) = 2/38 *Jinx*, 21/22 *Jinx* + ATCT, 7/7 *Jinx* + ATCT + challenge in ≥ 2 experiments).B, CBodyweight and virus titres in week 17 p.i. or postchallenge (*n* = 12 *Jinx*, 21 *Jinx* + ATCT, 7 *Jinx* + ATCT + challenge, 17 WT).DFrequency of transferred CD8 T cells in the spleens of recipients 1 day (*n* = 4), 3 weeks (*n* = 16) and 15 weeks (*n* = 21) after therapy or 15 weeks after challenge (*n* = 7). Data information: Horizontal lines in graphs represent mean values. Horizontal dashed line (C) indicates the detection limit. Data are mean ± SEM with *n* = 7–21 in ≥ 2 experiments. Statistics: log‐rank test (A), Mann–Whitney test (B, D), ***P ≤ 0.001; ****P ≤ 0.0001. Source data are available online for this figure.

### ATCT protects *Jinx* mice from HLH relapses by establishing long‐term donor chimerism

To evaluate whether ATCT protected long‐term from HLH relapses, *Jinx* mice were monitored over > 20 weeks. Twenty one of 22 *Jinx* mice with ATCT (4 × 10^6^ CD3^+^ T cells) survived, showed no signs of disease with normal bodyweight in week 17 p.i. and cleared LCMV (with residual virus in the kidney of some mice). By contrast, 36 of 38 *Jinx* mice without therapy had to be eliminated, exhibited significantly lower bodyweight, and were persistently infected (Fig 4A–C). Importantly, *Jinx* mice with successful ATCT were fully protected against HLH re‐induction after re‐challenging with LCMV (> 45 days after therapy) indicated by 100% survival, normal bodyweight 17 weeks after challenge, and complete elimination of LCMV (Fig 4A–C). Monitoring of donor cell chimerism after ATCT revealed an enormous expansion of therapeutic T cells in the spleen of infected *Jinx* hosts. While early CD8 T cell chimerism was around 0.25% 18 h after ATCT, therapeutic T cells expanded ~80‐fold within 3 weeks (Fig 4D) and persisted long‐term at high levels to protect from HLH relapses.

### T cell differentiation pattern as biomarker to predict successful ATCT in active HLH

**Figure 5:**
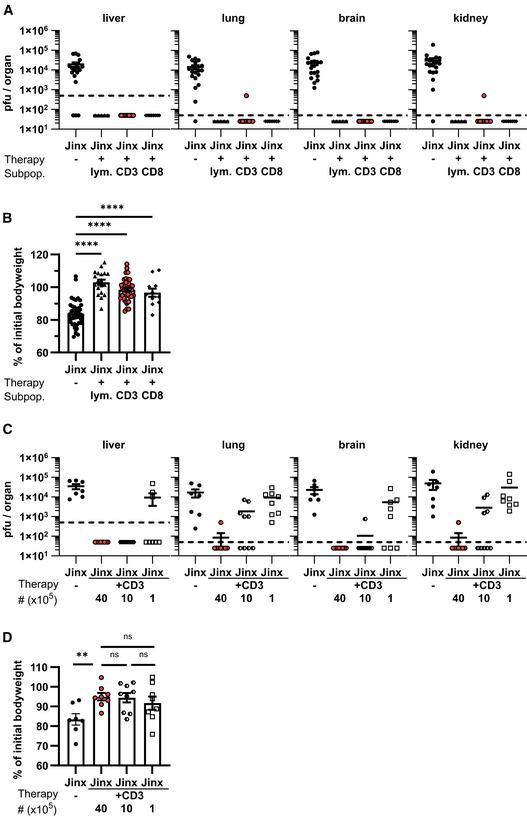
Successful ATCT does not strictly correlate with high bodyweight *Jinx* mice were infected with 200 pfu LCMV‐WE i.v.
A, BOn day 15 p.i., mice remained untreated or received 1 × 10^7^ total lymphocytes (*Jinx* + lym), 4 × 10^6^ purified CD3 T cells (*Jinx* + CD3), or 4 × 10^6^ purified CD8 T cells (*Jinx* + CD8) from LCMV‐immune wild‐type mice. (A) Virus titres (*n* = 19 *Jinx*, 5 *Jinx* + lymph, 16 *Jinx* + CD3, 8 *Jinx* + CD8) and (B) bodyweight on day 20 after therapy (*n* = 43 *Jinx*, 19 *Jinx* + lym, 42 *Jinx* + CD3, 11 *Jinx* + CD8).C, DOn day 15 p.i., *Jinx* mice remained untreated or received a transfer of 4 × 10^6^, 1 × 10^6^, or 1 × 10^5^ purified CD3 T cells from LCMV‐immune wild‐type mice. (C) Virus titres and (D) bodyweight on day 20 after therapy (*n* = 7 *Jinx*, 8 *Jinx* + 40 × 10^5^, 9 *Jinx* + 10 × 10^5^, 8 *Jinx* + 1 × 10^5^). Data information: Horizontal lines in graphs represent mean values. Horizontal dashed line (A, B) indicates the detection limit. Data are mean ± SEM with *n* = 5–19 per group in 1–8 independent experiments (A–D). Statistics: Mann–Whitney test (B, D), ns *P* > 0.05; **P ≤ 0.01; ****P ≤ 0.0001.

Requirements for curative ATCT were defined by the transfer of different lymphocyte populations and cell numbers. LCMV clearance by day 20 after therapy was chosen to determine therapeutic success since it correlated with the improved clinical status. Transfer of 1 × 10^7^ total lymphocytes or 4 × 10^6^ CD3 T cells or 4 × 10^6^ CD8 T cells from LCMV‐immune WT mice cleared virus in *Jinx* mice indicating cure of active HLH (Fig 5A and B). After ATCT with 4 × 10^6^ CD3 T cells 7/8 *Jinx* mice eliminated virus completely, 1 × 10^6^ transferred cells eliminated LCMV in 3/9 mice, whereas 1 × 10^5^ CD3 T cells were insufficient (Fig 5C). Bodyweight was unsuited to distinguish mice with successful from nonsuccessful ATCT due to strong individual variations (Fig 5B and D).

**Figure 6:**
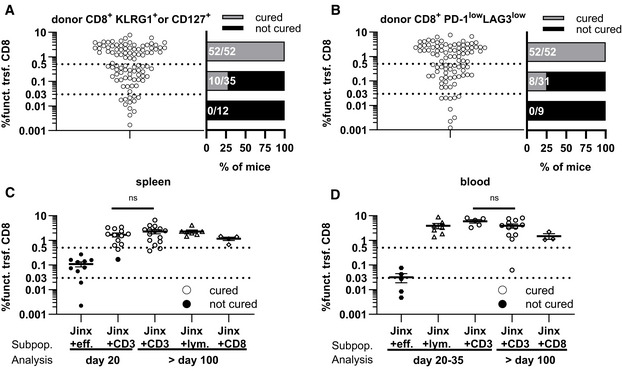
Frequency of adoptively transferred functional CD8 T cells in *Jinx* mice predicts therapy success A–D
*Jinx* mice were infected with 200 pfu LCMV‐WE i.v. Pooled data from ATCT experiments irrespective of the number and composition of the transferred cell population analyzed ≥ day 20 after therapy. (A) Frequency of “functional” transferred CD8 T cells (funct. trsf. CD8; KLRG1^+^ and/or CD127^+^) of all lymphocytes in the spleen was correlated with virus clearance to determine therapy success. Cured (LCMV‐free) recipients (gray bars) versus not cured (persistently infected) recipients (black bars; *n* = 99). (B) Procedure described in (A) was repeated for “functional” transferred CD8 T cells, which are PD‐1^low^LAG3^low^ (*n* = 92). (C, D) *Jinx* mice received on day 15 p.i. 1 × 10^7^ lymphocytes (*Jinx* + lym), 4 × 10^6^ purified CD3 (*Jinx* + CD3), or 4 × 10^6^ purified CD8 T cells (*Jinx* + CD8) from LCMV‐immune wild‐type mice. Alternatively, *Jinx* mice received effector T cells from acutely LCMV‐infected WT mice (day 5–15 p.i.; *Jinx* + eff). Frequency of transferred “functional” CD8 T cells (PD‐1^low^LAG3^low^ or CD127^+^ and/or KLRG1^+^) in recipient *Jinx* mice at the indicated time points after therapy in (C) spleen and (D) blood. Successful LCMV clearance from all organs (cured, open symbol) versus no LCMV clearance (not cured, filled symbol). (*n* (C) = 10 *Jinx* + eff. – day 20, 13 *Jinx* + CD3 – day 20, 15 *Jinx* + CD3 > day 100, 7 *Jinx* + lym. > day 100, 4 *Jinx* + CD8 > day 100). (*n* (D) = 5 *Jinx* + eff. – day 20–35, 7 *Jinx* + lym. – day 20–35, 5 *Jinx* + CD3 – day 20–35, 12 *Jinx* + CD3 > day 100, 3 *Jinx* + CD8 > day 100). Data information: Horizontal lines in graphs represent mean values. Dotted lines (A–D) indicate thresholds. Data are mean ± SEM with *n* = 92–99 in 18 independent experiments (A, B) and *n* = 5–19 in 1–8 independent experiments (C, D). Statistics: Mann–Whitney test (C, D), ns *P* > 0.05.

**Figure 7:**
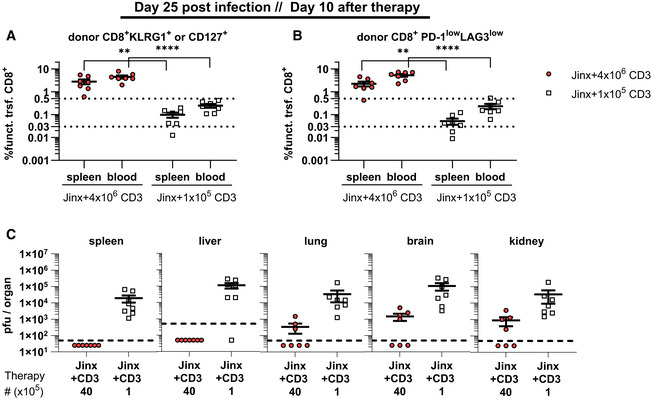
Frequency of adoptively transferred functional CD8 T cells in *Jinx* mice predicts therapy success 10 days post‐therapy *Jinx* mice were infected with 200 pfu LCMV‐WE i.v. On day 15 p.i., *Jinx* mice received a transfer of 4 × 10^6^ or 1 × 10^5^ purified CD3 T cells from LCMV‐immune wild‐type mice.
Frequency of “functional” transferred CD8 T cells (funct. trsf. CD8), (KLRG1^+^ and/or CD127^+^ of all lymphocytes) in recipients 10 days after therapy (*n* = 7 mice per group).Procedure of (A) was repeated for transferred CD8 T cells with low expression of PD‐1 and LAG3 (*n* = 7 mice per group).Virus titers were determined 10 days after therapy (*n* = 7 mice per group). Data information: Dotted lines (A, B) indicate thresholds. Horizontal lines in graphs represent mean values. Data are mean ± SEM with *n* = 7 mice per group in two experiments. Statistics: unpaired *t*‐test (A, B), ** *P* ≤ 0.01; **** *P* ≤ 0.0001.

**Figure EV4:**
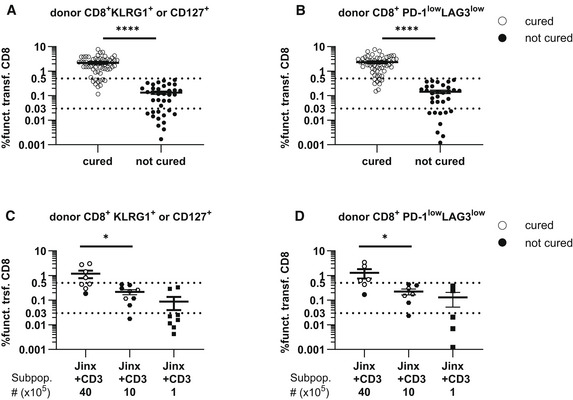
Frequency of adoptively transferred functional CD8 T cells in *Jinx* mice indicates therapy success 20 days post‐therapy *Jinx* mice were infected with 200 pfu LCMV‐WE intravenously.
A, BOn day 15 p.i., different numbers of lymphocytes, CD3, CD4, or CD8 T cells were transferred. Analyses ≥ day 20 after therapy. (A) Frequency of “functional” transferred CD8 T cells (funct. trsf. CD8; KLRG1^+^ and/or CD127^+^) of all lymphocytes in recipients that cleared LCMV (cured) or not (not cured; *n* = 62 “cured”, *n* = 37 “not cured” in 18 experiments). (B) Procedure of (A) was repeated for “functional” transferred CD8 T cells with low expression of PD‐1 and LAG3. (C, D) On day 15 p.i., *Jinx* mice remained untreated or received a transfer of 4 × 10^6^, 1 × 10^6^, or 1 × 10^5^ purified CD3 T cells from LCMV‐immune wild‐type mice (*n* = 60 “cured”, *n* = 32 “not cured” in 18 experiments).CFrequency of “functional” transferred CD8 T cells (funct. trsf. CD8), (KLRG1^+^ and/or CD127^+^ of all lymphocytes) in recipients that cleared LCMV (cured) or not (not cured) 20 days after therapy (*n* = 8–9 mice per group).DProcedure of (C) was repeated for transferred CD8 T cells with low expression of PD‐1 and LAG3 (*n* = 5–7 mice per group). Data information: Dotted lines (A–D) indicate thresholds. Horizontal lines in graphs represent mean values. Data are mean ± SEM with *n* (A, B) 32–62 mice in 18 experiments, *n* (C, D) 5–9 mice in 3 experiments. Statistics: Mann–Whitney test (A–D). ns *P* > 0.05; *P ≤ 0.05; ****P ≤ 0.0001. Source data are available online for this figure.

We hypothesized that KLRG1 and/or CD127 expression on transferred CD8 T cells reflected their functional efficacy and could serve as a predictive biomarker for therapeutic success and long‐term survival. Pooled data from all ATCT experiments irrespective of the number and composition of the transferred cell population were analyzed ≥ day 20 after therapy. When correlating the frequency of such “functional” transferred CD8 T cells in the spleen with virus titres as an indicator of therapy success, we were able to distinguish three response groups: (i) 100% therapeutic success (52 of 52 *Jinx* mice), if the frequency of therapeutic KLRG1 and/or CD127 expressing CD8 T cells in the splenic lymphocyte compartment was > 0.5%, (ii) partial therapeutic success (29%; 10/35 *Jinx* mice), if the frequency was between 0.03 and 0.5% and (iii) no therapeutic success (0/12 *Jinx* mice), if the frequency was < 0.03% (Figs 6A, and EV4A and C). We also evaluated the correlation of low expression of inhibitory receptors PD‐1/LAG3 on the therapeutic CD8 T cells: consistently, all *Jinx* mice with a frequency of > 0.5% of transferred, PD‐1^low^LAG3^low^ CD8 T cells were cured from active HLH (Figs 6B, and EV4B and D), demonstrating a strong correlation of both biomarkers. Independent of transferred lymphocyte populations and the time point of analysis a frequency of > 0.5% of “functional”, therapeutic CD8 T cells correlated with therapeutic success and long‐term survival (Fig 6C). Therapeutic failure of transferred effector T cells was reflected by frequencies < 0.5% on day 35. Of importance for clinical translation, similar results were obtained when analyzing blood samples (Fig 6D). This prompted us to evaluate whether successful ATCT might be predicted at an earlier time point by analyzing the frequency of functional transferred CD8 T cells. *Jinx* mice were infected and received on day 15 either 4 × 10^6^ or 1 × 10^5^ CD3 T cells as described but were analyzed on day 25, already 10 days after ATCT. Indeed, early analysis of biomarkers in blood and spleen predicted therapy success after the transfer of 4 × 10^6^ CD3 T cells at a time point when virus was not eliminated from all tissues (Fig 7A–C) and the clinical status was not yet improved (Fig 2A). In line, the biomarkers predicted nonsuccessful ATCT with 1 × 10^5^ CD3 T cells at this time point, with high virus load in all tissues.

**Figure 8:**
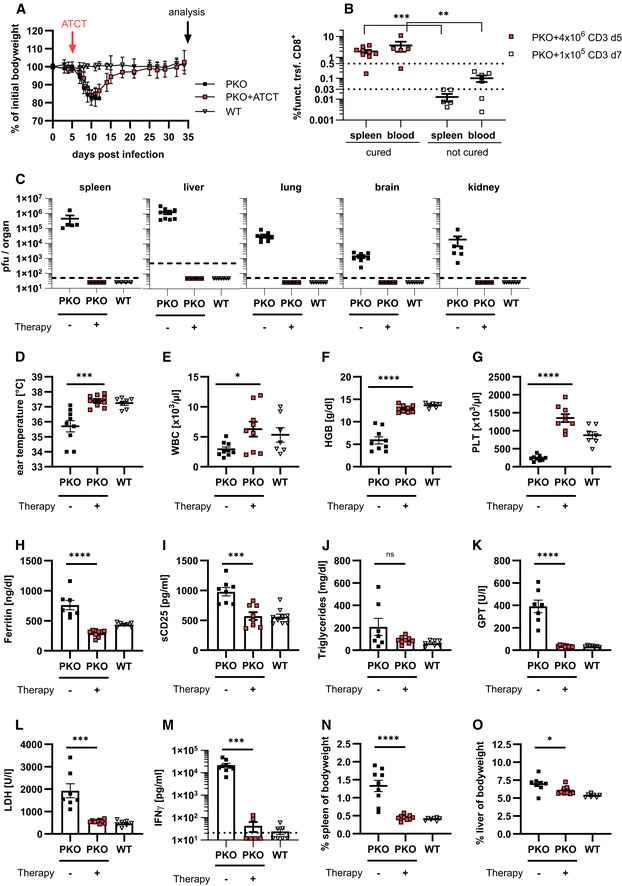
Successful adoptive T cell therapy in *Perforin*‐deficient mice *Perforin*‐deficient mice (PKO) and wild‐type controls (WT) were infected with 200 pfu LCMV‐WE i.v. On day 5 p.i. mice remained untreated (PKO, WT) or received 1 × 10^7^ lymphocytes or 4 × 10^6^ purified CD3 T cells from LCMV‐immune wild‐type mice (PKO + ATCT; pooled data). Untreated PKO mice were analyzed on day 12 p.i., PKO mice with transferred cells, and WT mice on day 25–30 after therapy.
ABodyweight of PKO, PKO + ATCT and WT mice for 5 weeks after infection (*n* = 9 PKO, 9 PKO + ATCT, 7 WT).BCell frequency of “functional” (PD‐1^low^LAG3^low^) transferred CD8 T cells in the spleen and blood of PKO + ATCT that eliminated LCMV (cured) compared with PKO recipients with insufficient therapy (transfer of 1 × 10^5^ purified CD3 T cells transferred on day 7 p.i., analysis on day 12–19 p.i.) that did not eliminate LCMV (not cured). (*n* (spleen) = 9 PKO + 4 × 10^6^, *n* = 6 PKO + 1 × 10^5^), (*n* (blood) = 5 PKO + 4 × 10^6^, *n* = 6 PKO + 1 × 10^5^).CVirus titres in liver, lung, brain, and kidney (*n* = 9 PKO, 9 PKO + ATCT, 7 WT).DEar temperature (*n* = 9 PKO, 9 PKO + ATCT, 7 WT).E–GFrequency of white blood cells (WBC), concentration of hemoglobin (HGB) and frequency of platelets (PLT) in blood (*n* = 9 PKO, 9 PKO + ATCT, 7 WT).H–MSerum concentration of ferritin, soluble CD25, triglycerides, glutamate‐pyruvate transaminase (GPT), lactate dehydrogenase (LDH) and interferon γ (IFNγ) (*n* = 7–9 PKO, 6–9 PKO + ATCT, 6–7 WT).N, OSpleen and liver weight were analyzed in relation to bodyweight of mice (*n* = 8–9 PKO, 9 PKO + ATCT, 7 WT). Data information: Dotted lines (B) indicate thresholds. Horizontal lines in graphs represent mean values. Data are mean ± SEM with *n* = 5–10 per group in 2–3 independent experiments. Statistics: unpaired *t*‐test (B–G, I–L, N), Mann–Whitney test (H, J, M, O), ns *P* > 0.05; *P ≤ 0.05; **P ≤ 0.01; ***P ≤ 0.001; ****P ≤ 0.0001.

### Predicting therapy success in *Perforin*‐deficient mice with ATCT

**Figure EV5:**
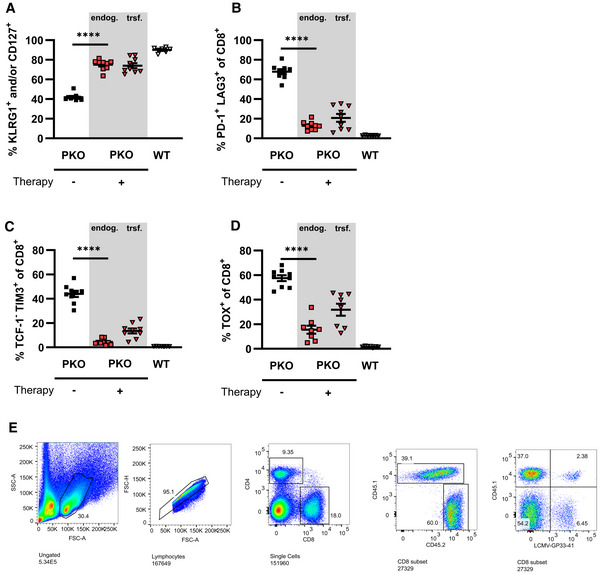
Readjusted T cell differentiation after adoptive T cell therapy in PKO mice Perforin‐deficient mice (PKO) and wild‐type controls (WT) were infected with 200 pfu LCMV‐WE intravenously. On day 5 post infection (p.i.), mice remained untreated (PKO, WT) or received an adoptive transfer of 1 × 10^7^ lymphocytes or 4 × 10^6^ purified CD3 T cells from LCMV‐immune wild‐type mice (PKO + ATCT). Transferred CD8 T cells (trsf.) were distinguished from endogenous CD8 T cells (endog.). Untreated PKO mice were analyzed on day 12 p.i., PKO mice with transferred cells, and WT mice on day 25–30 after therapy.
A–DEndogenous and transferred CD8 T cells were analyzed by flow cytometry: KLRG1^+^ and/or CD127^+^ (A), PD‐1^+^LAG3^+^ (B), TIM3^+^TCF‐1^−^ (C) and TOX^+^ (D) (*n* = 9 PKO, 9 PKO + ATCT, 7 WT). Data information: Data are mean ± SEM with *n* (A–D) 7–9 mice in 3 experiments. Statistics: Mann–Whitney test (A–D). ****P ≤ 0.0001.EExemplary gating strategy: (1) gating on lymphocytes; (2) exclusion of doublets; (3) determination of the frequency of CD4 and CD8 T cells, gating on CD8 T cells; (4) discrimination of CD45.1^+^ and CD45.2^+^ CD8 T cells; (5) analysis of CD45.1^+^ or CD45.2^+^ LCMV‐GP_33–41_‐specific CD8 T cells.

To confirm the robustness and validity of the predictive biomarkers, we performed ATCT experiments in PKO mice. These mice develop the first signs of disease at day 6 progressing into fatal HLH between day 10–20 after infection. Due to this rapid disease progression in PKO mice, intervention in ongoing HLH is not possible. ATCT was therefore performed on day 5, when viral immune stimulation was already well established, but clinical disease had not yet manifested. The goal was to prevent fatal HLH development. PKO mice receiving ATCT on day 5 lost bodyweight until day 10 but then regained weight and recovered completely around day 20 after therapy. Nontreated PKO mice fulfilling all HLH criteria by day 12 were eliminated to prevent loss of mice due to fatal HLH progression shortly thereafter (Fig 8A). Therapeutic success was reliably predicted by a frequency of > 0.5% of functional therapeutic CD8 T cells in blood or spleen of PKO mice (Fig 8B). By contrast, nonsuccessful therapy starting on day 7 with 1 × 10^5^ CD3 T cells from LCMV‐immune mice correlated with a frequency < 0.5% of functional CD8 T cells. PKO mice with successful ATCT starting at day 5 had cleared LCMV (Fig 8C) and recovered from all clinical HLH manifestations by day 30–35 p.i., when compared to diseased PKO mice analyzed by day 12 (Fig 8D–O). Ear temperature, hemoglobin, and platelets had normalized (Fig 8D–G). Ferritin, sCD25, GPT, and LDH had decreased significantly (Fig 8H–L). Serum levels of disease‐driving IFNγ were drastically reduced and hepatosplenomegaly improved significantly (Fig 8M–O). The therapeutic CD8 T cells retained a functionally active state with self‐renewal capacity as described for *Jinx* mice with ATCT (Fig EV5A–D). In summary, ATCT rescued PKO mice from lethal HLH progression. Importantly, therapeutic success could be reliably predicted by analyzing the frequency of “functional” therapeutic CD8 T cells in blood.

## Discussion

This study is the first proof‐of‐concept for successful ATCT in mouse models of FHL mice under conditions of hyperinflammation during active HLH. The treatment efficacy encourages to consider autologous trigger‐antigen‐experienced gene‐corrected T cells as an option in the treatment of patients with primary HLH not responding to current first‐line cytoreductive immunosuppressive interventions or suffering from HLH relapses.

At first glance, it is counterintuitive to enhance immunity under conditions of hyperinflammation to intervene in an active disease process. Therefore, we addressed three critical problems in this context: First, are the therapeutic T cells able to ameliorate the clinical HLH phenotype even when hyperinflammatory disease manifestations are already apparent? Second, are the therapeutic T cells able to remove the disease trigger by eliminating infected cells without aggravating the disease by further fuelling hyperinflammation? Third, can the endogenous, disease‐mediating T cells be silenced and thus removed from the disease process? We addressed these questions in preclinical models of HLH and demonstrate: (i) ATCT cured *Jinx* mice under conditions of hyperinflammation from active, virus‐triggered HLH without inducing life‐threatening side effects and protected PKO mice from lethal HLH progression. (ii) Donor T cells reconstituted the critical negative feedback loop on infected cells and eliminated the antigenic trigger. As a consequence, the endogenous, disease‐mediating T cells were no longer part of the disease process. (iii) Due to the enormous expansion of therapeutic T cells, ATCT established long‐term high‐level chimerism and prevented HLH relapses, even when mice were exposed to re‐challenging LCMV infection. Furthermore, we defined critical threshold frequencies of therapeutic T cells and a “functional” differentiation status as a biomarker to predict therapeutic success.

Notably, the use of autologous patient T cells for *ex vivo* gene correction and reinfusion does not require knowledge about the triggering agent as they will contain trigger‐antigen‐experienced T cells—whatever the trigger is. Previous studies have shown that gene‐corrected CD8 T cells from *Perforin*‐deficient or MUNC13‐4‐deficient mice protected mice prophylactically from LCMV‐triggered HLH (Carmo *et al*, 2015; Tiwari *et al*, 2016; Soheili *et al*, 2017; Ghosh *et al*, 2018; Takushi *et al*, 2020). Our proof‐of‐concept study demonstrating that ATCT is possible under conditions of hyperinflammation in HLH is an important prerequisite to further explore gene therapy approaches also for the treatment of ongoing disease. Some additional challenges remain when considering T cell gene therapy for clinical use in acute HLH: (i) the need to isolate T cells from a patient with hyperinflammation and cytopenia, (ii) the need for rapid immunosuppression that leaves a short window to harvest the T cells and (iii) the need to transduce hyperactivated T cells. As a first step to address one of these issues, successful *in vitro* gene therapy of hyperactivated T cells has been shown with PBMCs from MUNC13‐4 deficient patients with active HLH (Dettmer *et al*, 2019). Additional innovative approaches are under development for gene repair/editing of autologous primary human T cells to solve these problems and to restore functionality as demonstrated for FHL‐2, FHL‐3, XLP, IPEX, and CD40L (Hubbard *et al*, 2016; Ghosh *et al*, 2018; Panchal *et al*, 2018, 2021; Takushi *et al*, 2020; Borna *et al*, 2022).

Although we used a syngeneic setting, our findings may also have implications for therapeutic approaches based on adoptive transfer of allogeneic, partially HLA‐matched virus‐specific T cells (VST), as it is successfully used to prevent or treat virus infections/reactivations pre‐ and post‐HSCT (Leen *et al*, 2009; Heslop *et al*, 2010; Bollard & Heslop, 2016; Naik *et al*, 2016; Kaeuferle *et al*, 2019). More than 60 patients with IEI affecting virus control, have already received VST mainly post‐HSCT targeting active viral infections with high response rates (Creidy *et al*, 2016; Naik *et al*, 2016; Harris *et al*, 2019; Keller & Bollard, 2020). The therapeutic situation in patients with active HLH is more delicate than in patients with other inborn errors of immunity because the disease is associated with excessive hyperinflammation. Further studies will be necessary to evaluate VST rejection, long‐term survival, and compatibility with concurrent therapies in patients. However, our proof‐of‐concept study demonstrates that in principle, ATCT is also possible under the hyperinflammatory conditions of HLH. Since primary HLH patients initially receive substantial cytoreductive immunosuppressive therapy, rejection of transferred T cells may not represent a major problem. Several studies demonstrated that allogeneic virus‐specific T cells persist long enough to convey therapeutic efficacy in transfused hosts (Tzannou *et al*, 2017; Withers *et al*, 2017). Studies in HSCT patients with gene‐marked therapeutic T cells or analysis by TCR deep‐sequencing demonstrated that the expansion capacity of CD4 and CD8 T cell clonotypes, TCR diversity, and persistence of functional T cells correlate with the therapeutic efficacy of ATCT (Keller *et al*, 2019). Severe graft‐versus‐host disease (GVHD) is rarely observed due to the infusion of enriched VST, reducing the frequency of alloreactive T cells (Doubrovina *et al*, 2012; Tzannou *et al*, 2017). Indeed, enriched VST is being developed as an off‐the‐shelf product (Eiz‐Vesper *et al*, 2012; Papadopoulou *et al*, 2014; Tzannou *et al*, 2017) and might therefore be rapidly available for therapy of HLH patients with the virus‐triggered disease. It is obvious, that this approach requires knowledge of the viral trigger, which is the case in about 20% of primary HLH patients (Bergsten *et al*, 2017; Heeg *et al*, 2018).

We performed ATCT for active HLH without accompanying immunosuppression to detect potential toxic side effects of the treatment. Under these conditions, ATCT was extremely efficient and stopped disease progression within 20 days. Importantly, although infused during active hyperinflammation, the transferred T cells did not aggravate the disease by further fuelling the cytokine storm. This low incidence of complications in *Jinx* mice under syngeneic ATCT is in line with the low frequency of adverse events after allogeneic VST therapy in HSCT patients and contrasts the experience with CAR T cell therapy, which is associated with a cytokine‐release syndrome (CRS) quite frequently (Naik *et al*, 2016; Keller & Bollard, 2020; Greenbaum *et al*, 2021). It is tempting to speculate, that more efficient counter‐regulation in virus‐specific T cells, compared with engineered CAR T cells, is responsible for the less frequent complications. When translating our findings to patients, two critical points of ATCT in HLH have to be considered: First, does the initial anti‐inflammatory therapy interfere with the activity of the therapeutic T cells, and second, how to lower a potential risk of toxic side effects that are possibly not reflected in the mouse model? Thus, it will be important to control hyperinflammation by blocking cytokines and/or inhibiting distinct signaling pathways without affecting the activity of the therapeutic T cells and to target pathways to prevent potential therapy‐associated CRS. Recent studies using humanized mice that replicate CAR T cell‐induced CRS as observed in patients identified highly activated monocytes as producers of disease‐driving cytokines IL‐1 and IL‐6. Neutralization of these cytokines prevented CRS without affecting the function of CAR T cells (Giavridis *et al*, 2018; Norelli *et al*, 2018). ATCT based on VST from (partially) HLA‐matched donors has been successfully used to prevent or treat viral infections post‐HSCT (Leen *et al*, 2009; Heslop *et al*, 2010; Bollard & Heslop, 2016; Kaeuferle *et al*, 2019) or after solid organ transplantation (Roemhild & Reinke, 2016; Ivana *et al*, 2022) in patients not responding to standard antiviral therapies, even when performed in combination with cyclosporine A and moderate levels of corticosteroids (< 0.5 mg/kg/day). Tacrolimus and mycophenolate mofetil (MMF) have also been shown to be compatible with ATCT (Haque *et al*, 1998, 2001; Papadopoulou *et al*, 2014; Naik *et al*, 2016; Keller & Bollard, 2020).

Previous work using cell transfers in nondiseased HLH‐prone mice showed, that a threshold of 10–20% functional CD8 T cells is sufficient to protect PKO mice from LCMV‐triggered HLH (Terrell & Jordan, 2013b). The relevance of these findings was confirmed by studies of mixed chimerism in transplanted FHL patients (Hartz *et al*, 2016). We demonstrate, that an input number of memory T cells leading to a rather low initial frequency of ~0.25% in the CD8 T cell compartment (determined 18 h after transfer) was sufficient to generate a 5–30% chimerism within 3 weeks due to a ~80‐fold expansion of the therapeutic T cells. Whereas T cells from LCMV‐immune WT mice successfully cured *Jinx* mice from active HLH, transfer of T cells from naïve or acutely LCMV‐infected WT mice failed to stop disease progression illustrating the importance of the differentiation state of infused therapeutic T cells. This is in line with ATCT studies in patients and animal models showing that memory T cells, especially stem cell memory (Tscm) and central memory (Tcm), are superior to effector T cells in therapeutic efficacy (Kaech *et al*, 2002a, 2002b; Stemberger *et al*, 2009, 2014; Graef *et al*, 2014; Neuenhahn *et al*, 2017). These memory T cells are characterized by high proliferative potential, broad reconstitution/differentiation capacity, and long persistence, which seems to be a prerequisite for therapeutic success. Accordingly, subpopulations of T cells isolated from patients with HLH and undergoing *ex vivo* gene therapy have to be selected for these properties.

We identified a marker combination (based on distinct KLRG1/CD127 or PD‐1/LAG‐3 expression patterns) reflecting a “functional”, nonexhausted state of therapeutic T cells in the treated recipients that correlated with therapeutic success. Interestingly, the “functional” murine T cells also expressed the transcription factor TCF‐1, which is associated with self‐renewal capacity, supporting long‐term persistence. Independent of composition and number of transferred T cells, the time point of analysis (from 20–100 days after transfer), as well as the preclinical mouse model used, all mice with a frequency of > 0.5% “functional” therapeutic T cells in the total lymphocyte compartment were cured from active HLH. This observation suggests that numeric and phenotypic monitoring of therapeutic T cells during ATCT of patients can be highly valuable to predict therapy success. Importantly, the phenotype of the disease‐driving CD8 T cells in mice during active HLH resembles those of patients with untreated HLH, reflected by strong downregulation of CD127 on most of the cells (Ammann *et al*, 2017), a prominent KLRG1/CD127 double negative population and a population with high expression of inhibitory receptor PD‐1. In line with our results, high PD‐1 expression with partial co‐expression of the inhibitory receptors LAG3 or TIM3 has been shown for CD8 T cells in 1°HLH patients (Kelkar *et al*, 2021; Kumar *et al*, 2022). Thus, the marker combination used to determine “functional” T cells in this study to predict therapy success in the preclinical mouse models can presumably be directly transferred to HLH patients.

In summary, as a proof‐of‐concept, we have validated ATCT to successfully intervene in a clinical situation of severe virus‐triggered hyperinflammation. ATCT cured mice from active HLH by silencing the disease process without severe side effects and protected long‐term from HLH relapses and disease re‐induction with the same trigger. Given the promising results seen in the treatment of active HLH in the mouse models, our study may pave the way for the adoptive transfer of *ex vivo* gene‐corrected autologous T cells as an additional future therapeutic option to be considered for patients with refractory HLH.

## Materials and Methods

### Antibody list

**Table:** 

Antigen	Clone	Fluorochrome	Dilution 1	Supplier name
CD8a	53–6.7	BV510/PerCPCy5.5/BV711	200/400/1,000	Biolegend
CD4	RM4‐5	BV650	1,000	Biolegend
CD90.1 (Thy1.1)	HIS51	AF488/APC/APCeFluor780	1,000/1,500/1,000	Thermo Fisher Scientific
CD90.2 (Thy1.2)	30‐H12	APC/BV510	600/150	Biolegend
CD45.1	A20	AF700/APC‐Fire750	200/200	Biolegend
CD45.2	104	AF488/AF700/BV785	200/200/100	Biolegend
CD44	IM7	APC/AF488	1,000/200	Thermo Fisher Scientific
CD62L	MEL‐14	BV650/BV510	1,500/400	Biolegend
KLRG1	2F1	PerCPCy5.5	150	Thermo Fisher Scientific
CD127	A7R34	BV421	100	Biolegend
CD3e	145‐2C11	PE‐Cy7	300	Biolegend
PD‐1 (CD279)	J43	BV785/PerCPCy5.5/APC	300/400/100	Thermo Fisher Scientific
LAG‐3(CD223)	C9B7W	PE‐Cy7	100	Biolegend
IFNγ	XMG1.2	APC/APC‐Fire750/BV510	250/250/50	Biolegend
TNFα	MP6‐XT22	AF488/PE	100/200	Biolegend
CD107a	1D4B	PE/AF488	50/50	Biolegend
TCF‐1/TCF‐7	C63D9	AF488/APC	100/100	Cell Signaling
TOX	TXRX10	APC	100	Thermo Fisher Scientific
CX3CR1	SA011F11	BV785/PE	1,000/200	Biolegend
CD366 (TIM3)	RMT3‐23	BV785/APC	100	Biolegend
Dead cells	Zombie	APC‐Cy7	1,000	Biolegend

### Study design

The first research objective of the controlled laboratory experiments was the analysis of the HLH disease course of *Jinx* mice later than 2 weeks post infection with the HLH trigger, LCMV‐WE. Second, the efficacy of an ATCT was planned to be determined in *Jinx* mice. After having established the ATCT in *Jinx* mice and analyzed the data, the third objective was the establishment of ATCT in PKO mice. When mice receiving ATCT were chosen on the day of ATCT, the formation of treatment groups was assigned so that the bodyweight distribution on the day of transfer was equally in nontreated control mice and mice receiving ATCT.

### Mice, LCMV infection

Male and female mice were used for this study and experiments were conducted according to the ARRIVE guidelines. Mice were eliminated, if they lost > 25% of their initial bodyweight on two consecutive days or if they showed apathy or neurological failures. Mice were kept under specific pathogen‐free conditions. *Jinx* (C57BL/6J‐Unc13dJinx/Mmucd) mice were purchased from MMRRC and bred with *Unc13D* ^*WT/Jinx*^ littermates. PKO (C57BL/6‐Prf1^tm1Sdz^) mice were obtained from Dr. Hengartner, Zurich. WT mice (C57BL/6N) were purchased from Janvier, France. Ear temperature was measured with ThermoScan 6022 (BRAUN). Mice were infected with LCMV‐WE (Dr. Lehmann‐Grube, Hamburg) or LCMV‐Armstrong (Dr. Zinkernagel, Zurich). LCMV was quantified using a focus‐forming assay (Battegay *et al*, 1991).

### T cell isolation and adoptive transfer

Untouched T cell isolation of CD3 or CD8 T cells (MojoSort, Biolegend) was performed according to the manufacturer's instructions prior to adoptive transfer in FCS‐free IMDM by i.v. injection in the tail vein (300 μl injection volume). Oat flakes were added daily to the nutrition of *Jinx*/PKO mice with and without ATCT for ≤ 10 days after therapy start.

### Patient material

Only samples were used that had been collected in a previous study. Previously described 1°HLH patient samples (Ammann *et al*, 2017) were re‐analyzed. We re‐analyzed eight FHL‐2 patients, seven FHL‐3 patients, and two FHL‐5 patients. As described by the authors in Ammann *et al* (2017), patients were recruited to this project through the HLH study of the German Society of Pediatric Hematology and Oncology. Control samples from healthy donors under 1 year of age were obtained from a clinic of HIV‐exposed (but not infected) infants. Informed consent was obtained from all subjects (including healthy donors) and the experiments conformed to the principles set out in the WMA Declaration of Helsinki and the Department of Health and Human Services Belmont Report.

### Histology

HE‐staining was performed as previously described (Institute of Surgical Pathology), analyzed at Carl Zeiss AxioImager M1 microscope (10× objectives) using Axiocam‐506 with ZEN core v2.7 software (Zeiss; Kögl *et al*, 2013).

### Blood and serum analyses

Blood analysis was performed by Sysmex KX‐21 hematology analyzer. Serum levels of glutamine‐pyruvate transaminase (GPT), lactate dehydrogenase (LDH), ferritin, and triglycerides were quantified using Roche Modular Analytics Evo. Soluble CD25 in serum was determined using IL‐2Ralpha DuoSet kit (R&D Systems), and serum IFNγ was determined with mouse IFN‐gamma ELISA MAX (Biolegend).

### Flow cytometry

Antibodies were purchased from BD Biosciences, Biolegend, and eBioscience/Thermo Fisher, stainings performed ≥ 30 min at 4°C. For intranuclear detection of transcription factors eBioscience FoxP3‐Staining‐Buffer‐Set (Invitrogen) was used. Detection of cytoplasmic cytokines was performed as previously described (Kögl *et al*, 2013). LCMV‐specific CD8 T cells were detected with fluorochrome‐labeled H‐2D^b^ tetramers (in‐house production and Tetramer Core Facility, Baylor College of Medicine). Gating strategy is depicted in Fig EV5E.

### Study approval

Mouse experiments were approved by Regierungspräsidium Freiburg (G‐16/06, G‐17/99, G‐18/125).

Acquired data from previously described 1°HLH patient samples were re‐analyzed (Ammann *et al*, 2017). As described by the authors, the study with patient samples was carried out after obtaining institutional review board approval (University of Freiburg ethics committee's protocol numbers 143/12 and 40/08 for patients and ethics number 282/11 for HD).

### Statistical analysis

Planned sample size was *n* = 10 per experimental group (Power 80%, standardized effect size 0.30); *n* > 10 reflects pooled data of different experimental questions and/or analyses at different time points. Statistical tests (one‐way ANOVA comparing multiple samples followed by unpaired *t*‐test comparing two samples; log‐rank‐test for survival, Mann–Whitney test for samples that were not normally distributed according to normality tests) were conducted using GraphPad Prism 8/9. No outliers were excluded. Exact *P*‐values for all statistical tests are available in the Appendix.

## Author contributions

**Kristoffer Weißert:** Conceptualization; formal analysis; validation; investigation; visualization; methodology; writing – original draft; writing – review and editing. **Sandra Ammann:** Conceptualization; formal analysis; investigation; visualization; writing – original draft; writing – review and editing. **Tamara Kögl:** Conceptualization; formal analysis; investigation. **Viviane Dettmer‐Monaco:** Resources; writing – review and editing. **Christoph Schell:** Formal analysis; investigation; writing – review and editing. **Toni Cathomen:** Funding acquisition; project administration; writing – review and editing. **Stephan Ehl:** Conceptualization; funding acquisition; writing – original draft; writing – review and editing. **Peter Aichele:** Conceptualization; supervision; funding acquisition; validation; visualization; writing – original draft; project administration; writing – review and editing.

## Disclosure and competing interests statement

The authors declare that they have no conflict of interest.

## Supporting information



Appendix

Expanded View Figures PDF

Source Data for Expanded View

Source Data for Figure 4

PDF+

## Data Availability

This study includes no data deposited in external repositories.
