# Supplementary material for: Adoptive T cell therapy cures mice from active hemophagocytic lymphohistiocytosis (HLH)
Source: EMBO Mol Med. 2022 Oct 24;14(12):e16085. doi: 10.15252/emmm.202216085 (PMC9728053; doi:10.15252/emmm.202216085)
Supplement: Supplementary file 1 — Appendix [file EMMM-14-e16085-s004.pdf]

**Appendix**	**page**
Appendix Table S1	1-3

Appendix Table S1: exact p-values

**Figure 1**	**Jinx vs. WT**			
D	<0.0001			
E	<0.0001			
F	<0.0001			
H	<0.0001			
I	0.0382			
J	<0.0001			
K	<0.0001			
L	<0.0001			
M	<0.0001			
N	0.0525			
O	<0.0001			
P	<0.0001			
Q	<0.0001			
**Figure 2**	**Jinx vs. Jinx + ATCT**	**Jinx + ATCT vs. WT**	**Jinx vs. WT**	
C	0.183	0.0363		
D	0.476	0.015		
E	0.092	0.0178		
F	0.0109	0.3385	0.0024	
G	<0.0001	0.0292		
H	0.6334	0.7802		
I	0.7758	0.0519	0.0901	
J	0.001	0.6506		
K	0.0377	0.9136	0.0467	
L	<0.0001			
M	0.0009	<0.0001		
N	<0.0001	0.0029		
**Figure 3**	**Jinx vs. Jinx + ATCT**			
	**CD8 T cells**	**GP33-specific**	**>d100 after therapy**	
A	<0.0001	<0.0001	0.056	
B	<0.0001	<0.0001	0.7401	
C	<0.0001	<0.0001	0.042	
**Figure 4**	**Jinx vs. Jinx + ATCT**			
A	<0.0001			
B	<0.0001			
D	0.0004			

**Figure 5**	**Jinx vs. Jinx+lym.**	**Jinx vs. Jinx+CD3**	**Jinx vs. Jinx+CD8**	
B	<0.0001	<0.0001	<0.0001	
	**Jinx vs. Jinx+40x10^5 CD3**	**Jinx+40x10^5 CD3 vs. Jinx+10x10^5 CD3**	**Jinx+10x10^5 CD3 vs. Jinx+1x10^5 CD3**	**Jinx+40x10^5 CD3 vs. Jinx+1x10^5 CD3**
D	0.0059	0.8148	0.743	0.5054
**Figure 6**				
C	**Jinx+CD3 day 20 vs. Jinx+CD3 >day 100**			
	0.413			
D	**Jinx+CD3 day 20 vs. Jinx+CD3 >day 100**			
	0.1681			
**Figure 7**				
	**Jinx+40x10^5 CD3 vs. Jinx+1x10^5 CD3 in spleen**	**Jinx+40x10^5 CD3 vs. Jinx+1x10^5 CD3 in blood**		
A	0.0018	<0.0001		
B	0.0015	<0.0001		
**Figure 8**	**PKO vs. PKO + ATCT**			
D	0.0006			
E	0.0203			
F	<0.0001			
G	<0.0001			
H	<0.0001			
I	0.0007			
J	0.1181			
K	<0.0001			
L	0.0002			
M	0.0004			
N	<0.0001			
O	0.0385			
**Figure EV1**				
A	<0.0001			
B	<0.0001			
C	<0.0001			
D	<0.0001			

**Figure EV3**				
	**Jinx vs. Jinx + ATCT**			
	**CD8 T cells**	**GP33-specific**	**>d100 after therapy**	
A	<0.0001	0.0005	0.0258	
B	0.0012	0.0043	0.0106	
C - IFNgTNFa	0.1078		0.1905	
C - IFNgCD107a	0.5237		0.381	
**Figure EV4**				
A	<0.0001			
B	<0.0001			
	**Jinx+40x10^5 CD3 vs. Jinx+10x10^5 CD3**			
C	0.0232			
D	0.0221			
**Figure EV5**				
	**PKO vs. PKO + ATCT**			
A	<0.0001			
B	<0.0001			
C	<0.0001			
D	<0.0001			
