# Supplementary material for: Adoptive T cell therapy cures mice from active hemophagocytic lymphohistiocytosis (HLH)
Source: EMBO Mol Med. 2022 Oct 24;14(12):e16085. doi: 10.15252/emmm.202216085 (PMC9728053; doi:10.15252/emmm.202216085)
Supplement: Supplementary file 2 — Expanded View Figures PDF [file EMMM-14-e16085-s001.pdf]

Expanded View Figures

Figure EV1. CD8 T cell phenotype in HLH mouse models and FHL patients.

Left column: CD8 T cells obtained from 1°HLH patients (1°HLH) were analyzed by flow cytometry in comparison to healthy donors (HD). Right column: *Jinx* and PKO mice (1°HLH) were infected with 200 pfu LCMV-WE intravenously and analyzed by flow cytometry on day 12–15 p.i. in comparison to noninfected wild-type mice (WT).

A, B The frequency of CD8 T cells double negative for KLRG1 and CD127 ($KLRG1^{-}CD127^{-}$) was determined (A) in blood of 1°HLH patients, HD and (B) in the spleen of 1°HLH mouse models, as well as noninfected WT mice (n = 17 1°HLH patients, n = 11 HD, n = 30 1°HLH mice, n = 13 WT mice).

C, D Frequency of CD8 T cells expressing PD-1 was determined (C) in blood of 1°HLH patients in comparison to HD (D) and in the spleen of 1°HLH mouse models, as well as noninfected WT mice (n = 17 1°HLH patients, n = 11 HD, n = 21 1°HLH mice, n = 11 WT mice).

E Expression of CD45RA and CCR7 on CD8 T cells was determined in blood of 1°HLH patients and HD and four populations were distinguished: $CD45RA^{+}CCR7^{+}$ termed "naïve", $CD45RA^{-}CCR7^{+}$ termed "T_{CM}", $CD45RA^{-}CCR7^{-}$ termed "T_{EM}" and $CD45RA^{+}CCR7^{-}$ (n = 17 1°HLH patients, n = 11 HD).

F Expression of CD62L and CD44 on CD8 T cells was determined in the spleen of 1°HLH mouse models, and noninfected WT mice and four populations were distinguished: $CD44^{-}CD62L^{+}$ termed "naïve", $CD44^{+}CD62L^{+}$ termed "T_{CM}", $CD44^{+}CD62L^{-}$ termed "T_{EM}" and $CD44^{-}CD62L^{-}$ (n = 19 1°HLH mice, n = 9 WT mice).

Data information: Horizontal lines in graphs represent mean values. Data are mean ± SEM. Statistics: unpaired t-test (B, D), Mann–Whitney test (A, C). ****$P \leq 0.0001$. Source data are available online for this figure.

 © 2022 The Authors

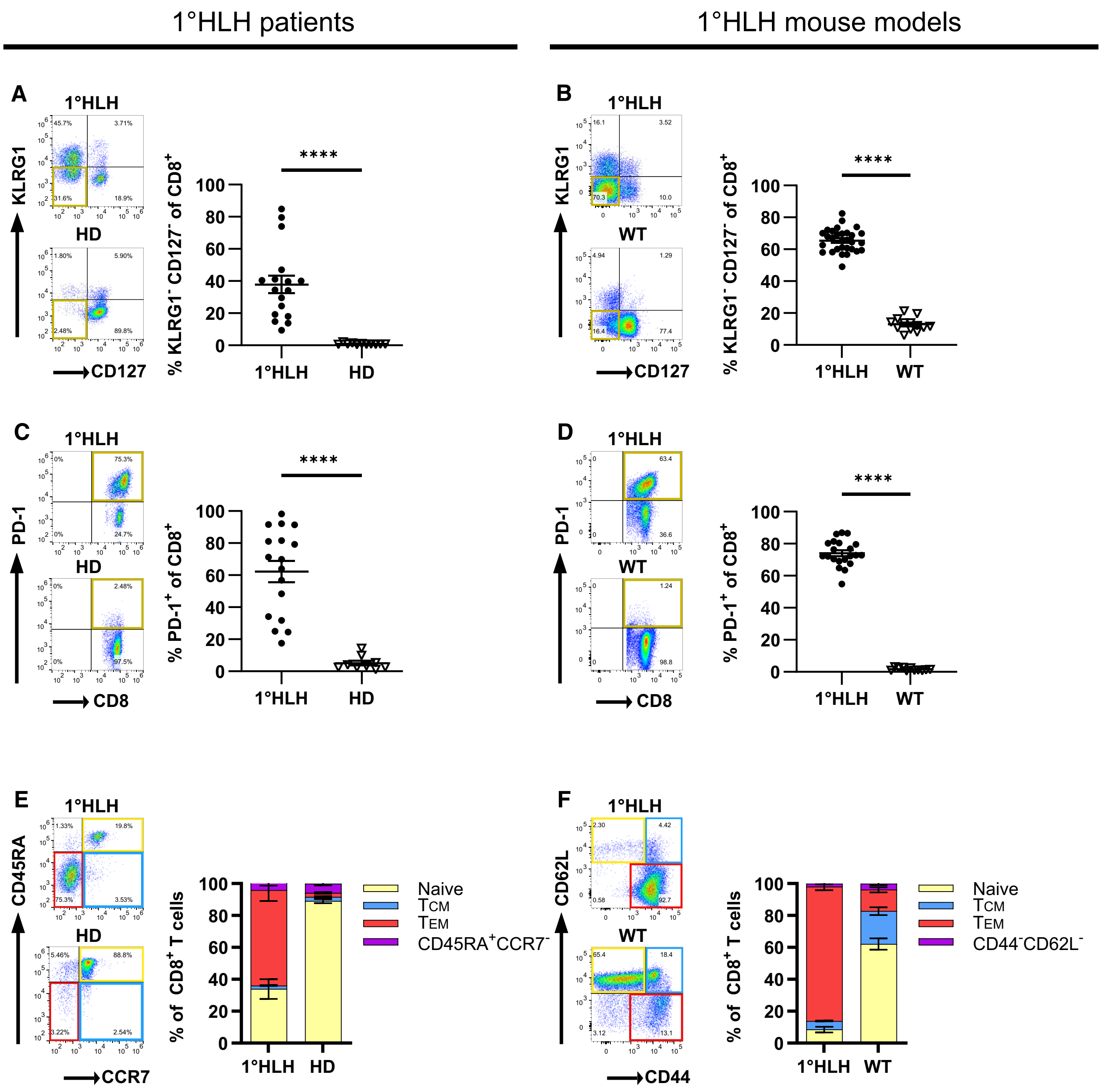

Figure EV1.

© 2022 The Authors

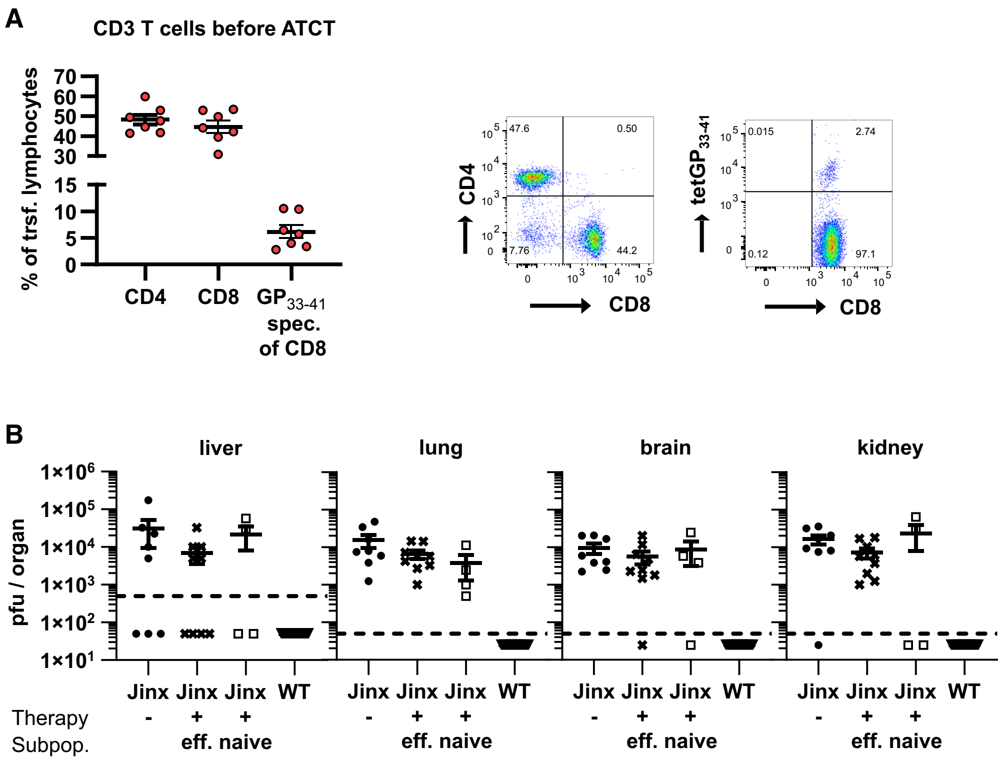

Figure EV2. Adoptive T cell therapy with naïve or LCMV-effector T cells in active HLH is not successful.

A Purified CD3 T cells used for ATCT were analyzed regarding the frequency of CD4 and CD8 T cells and the frequency of LCMV-$GP_{33–41}$-specific CD8 T cells among CD8 T cells. Individual dots represent separate transfers/experiments (*n* = 7).

B *Jinx* mice (*Jinx*) and heterozygous littermates (WT) were infected with 200 pfu LCMV-WE intravenously. On day 5–15 p.i., mice remained untreated (*Jinx*, *n* = 8; WT, *n* = 10) or 1 × 10^7 purified CD3 T cells/lymphocytes were transferred to *Jinx* mice (*Jinx* + eff, *n* = 9) Alternatively, *Jinx* mice received on day 15 p.i. a transfer with 1 × 10^7 CD3 T cells from uninfected wild-type mice (*Jinx* + naïve, *n* = 4).

Data information: Horizontal lines in graphs represent mean values. Data are mean ± SEM with *n* = 4–10 mice in 1–3 experiments.

Figure EV3. Expression of transcription factors and functionality of T cells after adoptive T cell therapy in active HLH.

Jinx mice (*Jinx*) and heterozygous littermates (WT) were infected with 200 pfu LCMV-WE intravenously. On day 15 postinfection (p.i.), mice remained untreated (*Jinx*, WT) or 4 × 10^6 purified CD3 T cells from LCMV-immune WT mice were transferred to *Jinx* mice (Jinx + ATCT). Transferred CD8 T cells (trsf.) were distinguished from endogenous CD8 T cells (endog.).

A, B On day 20 after therapy, endogenous and transferred CD8 T cells (column II), as well as LCMV-$GP_{33–41}$-specific CD8 T cells (column III), were analyzed by flow cytometry: frequency of TCF-1^+ or TOX^+. The same analyses were performed more than 100 days after therapy (column IV).

C Splenocytes were restimulated with LCMV-$GP_{33–41}$. The frequencies of transferred WT CD8 T cells in *Jinx* recipients and WT CD8 T cells secreting IFNγ and TNFα or IFNγ and CD107a after restimulation were determined on day 20 or > 100 days after therapy start (C, columns II, IV).

Data information: Horizontal lines in graphs represent mean values. ns $P > 0.05$; *$P \leq 0.05$; **$P \leq 0.01$; ***$P \leq 0.001$; ****$P \leq 0.0001$. Data are mean ± SEM with *n* (A–C) 3–19 mice in 1–5 experiments. Statistics: unpaired *t*-test (A column II, III, IV; B column IV), Mann–Whitney test (B column II, III; C column II, IV). Detailed information *n*: A. (II) *n* = 9 *Jinx*, 11 *Jinx* + ATCT (9× trsf. cells), 11 WT in 4 experiments; (III) *n* = 4 *Jinx*, 5 *Jinx* + ATCT (3× trsf. cells), 6 WT in 2 experiments; (IV) *n* = 10 *Jinx* + ATCT, 3 WT in 2 experiments. B. (II) *n* = 9 *Jinx*, 11 *Jinx* + ATCT (6× trsf. cells), 11 WT in 4 experiments; (III) *n* = 5 *Jinx*, 6 *Jinx* + ATCT (3× trsf. cells), 7 WT in 2 experiments; (IV) *n* = 9 *Jinx* + ATCT (6× trsf. cells), 3 WT in 2 experiments. C. (II) IFNγ/TNFα: *n* = 16 trsf. Cells in *Jinx* + ATCT, 16 WT in 5 experiments; (II) IFNγ/CD107a: *n* = 11 trsf. Cells in *Jinx* + ATCT, 10 WT in 3 experiments; (II) IFNγ/TNFα: *n* = 6 trsf. Cells in *Jinx* + ATCT, 3 WT in 1 experiment; (IV) IFNγ/CD107a: *n* = 6 trsf. Cells in *Jinx* + ATCT, 3 WT in 1 experiment.

 © 2022 The Authors

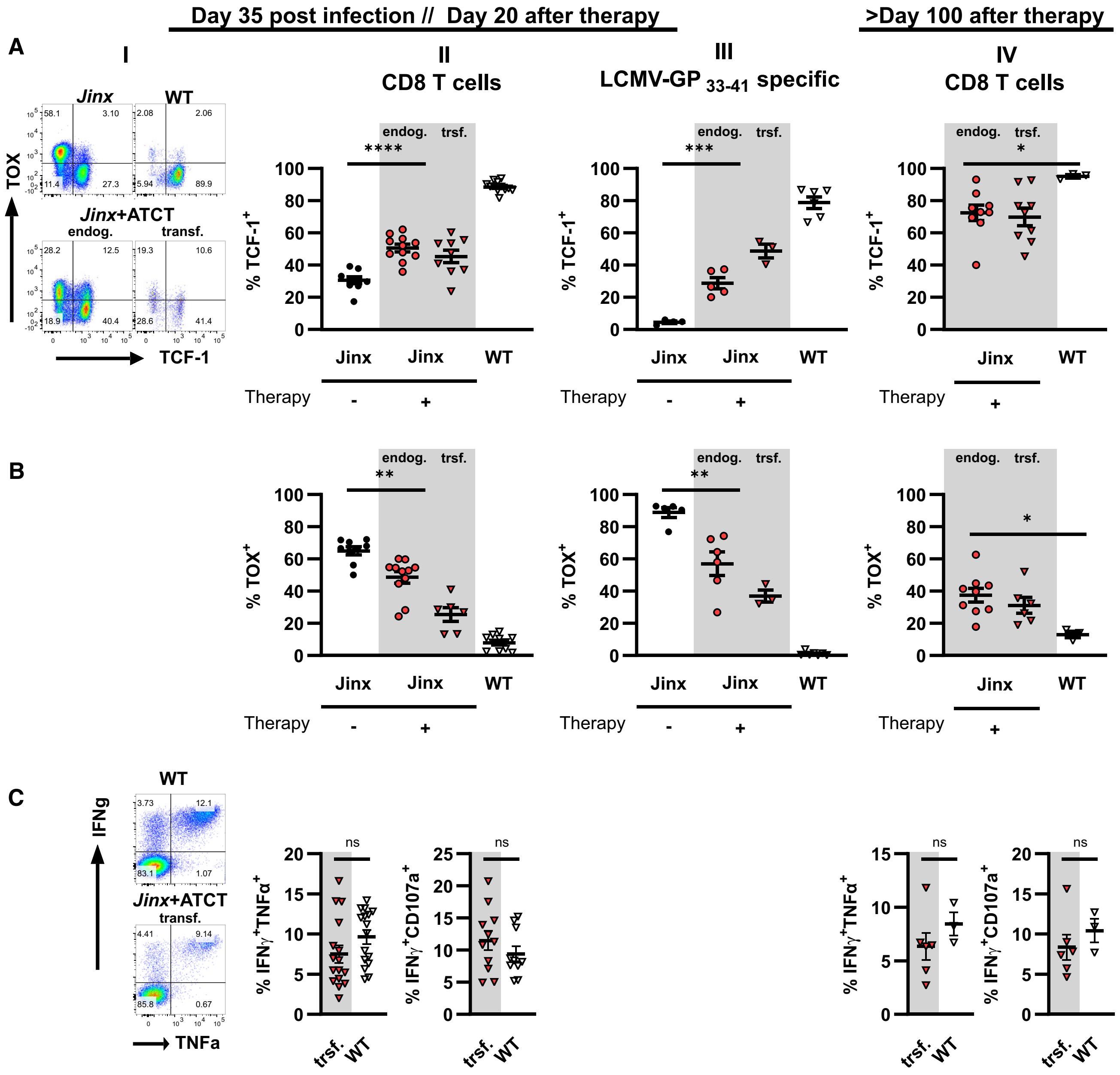
Day 35 post infection // Day 20 after therapy
>Day 100 after therapy
A
I
II
CD8 T cells
III
LCMV-GP 33-41 specific
IV
CD8 T cells
Jinx
WT
Jinx+ATCT
endog.
transf.
TOX
TCF-1
endog.
trsf.
% TCF-1+
Jinx
Jinx
WT
Therapy
-
+
B
% TOX+
C
WT
IFNg
TNFa
ns
% IFNγ+TNFα+
% IFNγ+CD107a+
trsf.
WT

Figure EV3.

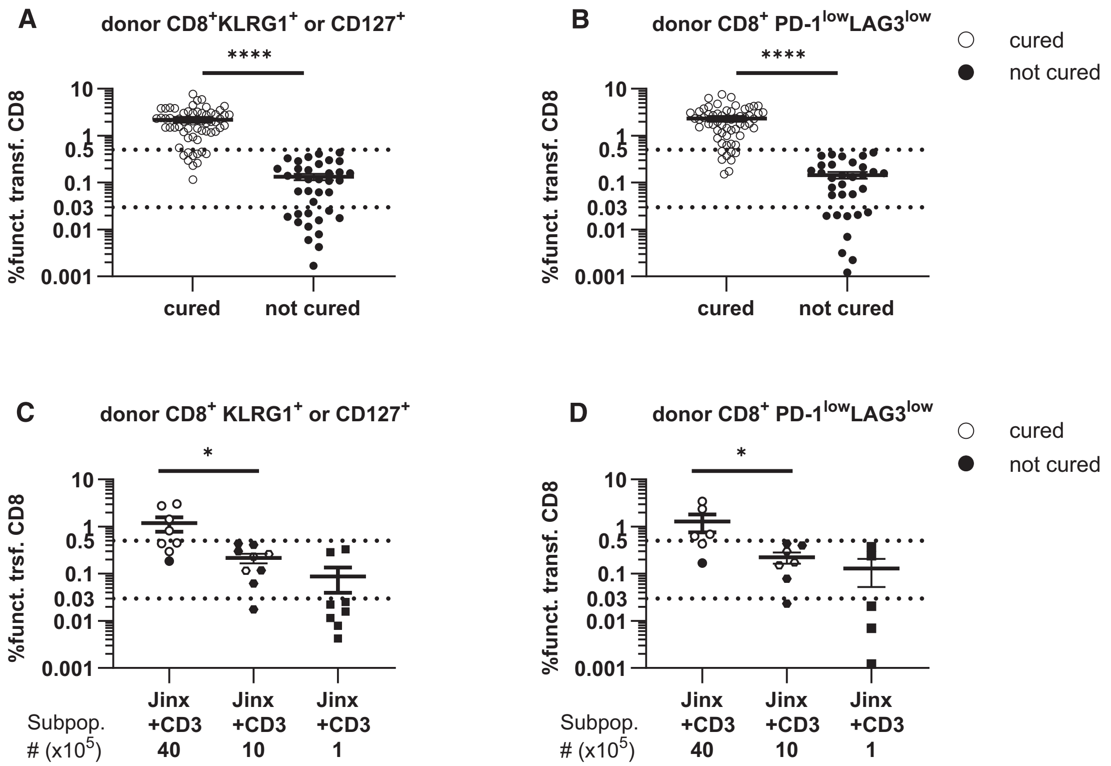

Figure EV4. Frequency of adoptively transferred functional CD8 T cells in *Jinx* mice indicates therapy success 20 days post-therapy.

Jinx mice were infected with 200 pfu LCMV-WE intravenously.

A, B On day 15 p.i., different numbers of lymphocytes, CD3, CD4, or CD8 T cells were transferred. Analyses ≥ day 20 after therapy. (A) Frequency of "functional" transferred CD8 T cells (funct. Trsf. CD8; KLRG1$^+$ and/or CD127$^+$) of all lymphocytes in recipients that cleared LCMV (cured) or not (not cured; n = 62 "cured", n = 37 "not cured" in 18 experiments). (B) Procedure of (A) was repeated for "functional" transferred CD8 T cells with low expression of PD-1 and LAG3. (C, D) On day 15 p.i., Jinx mice remained untreated or received a transfer of 4×10^6, 1×10^6, or 1×10^5 purified CD3 T cells from LCMV-immune wild-type mice (n = 60 "cured", n = 32 "not cured" in 18 experiments).

C Frequency of "functional" transferred CD8 T cells (funct. Trsf. CD8), (KLRG1$^+$ and/or CD127$^+$ of all lymphocytes) in recipients that cleared LCMV (cured) or not (not cured) 20 days after therapy (n = 8–9 mice per group).

D Procedure of (C) was repeated for transferred CD8 T cells with low expression of PD-1 and LAG3 (n = 5–7 mice per group).

Data information: Dotted lines (A–D) indicate thresholds. Horizontal lines in graphs represent mean values. Data are mean ± SEM with n (A, B) 32–62 mice in 18 experiments, n (C, D) 5–9 mice in 3 experiments. Statistics: Mann–Whitney test (A–D). ns $P > 0.05$; *$P \leq 0.05$; ****$P \leq 0.0001$.
Source data are available online for this figure.

 © 2022 The Authors

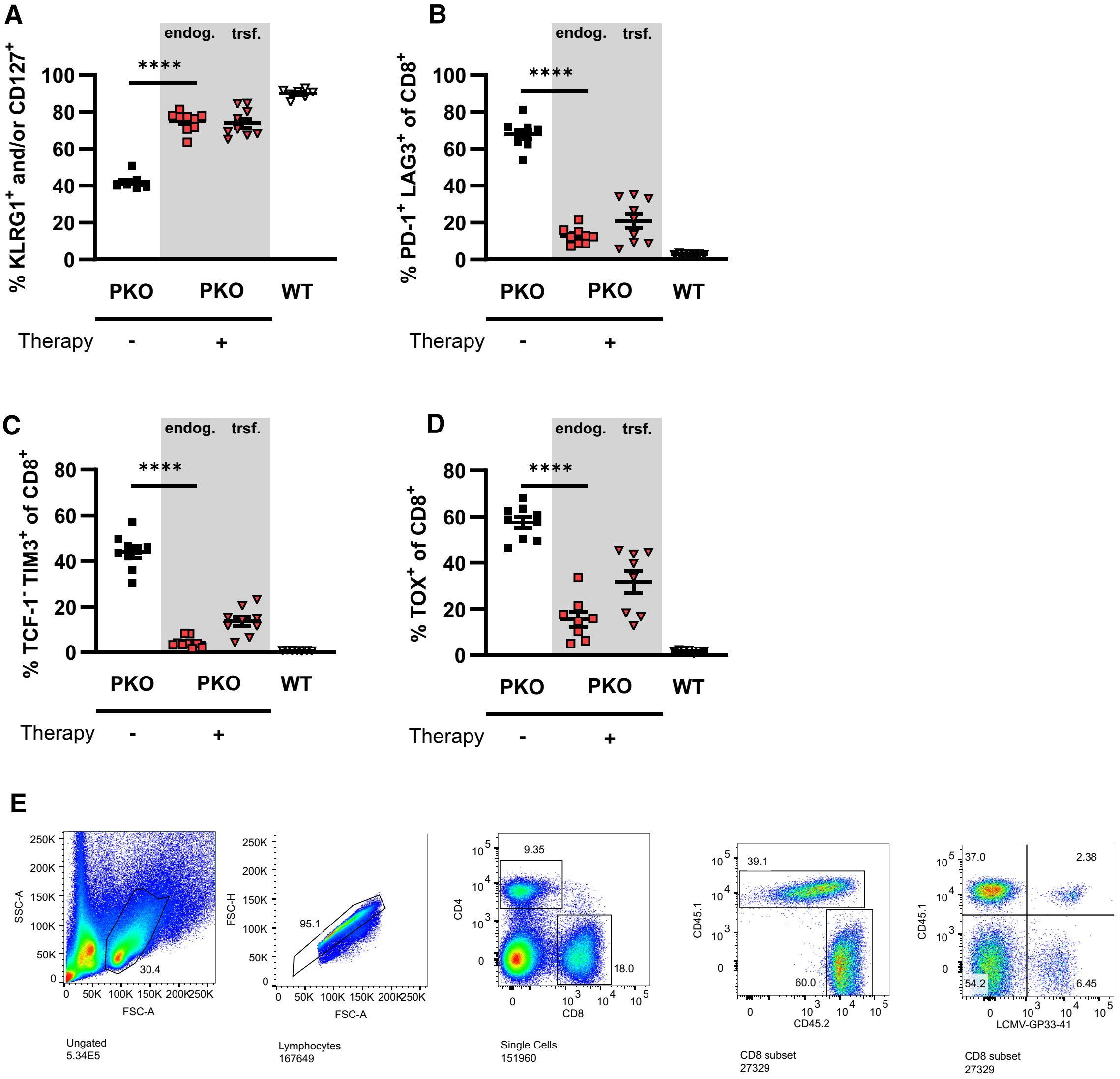

Figure EV5. Readjusted T cell differentiation after adoptive T cell therapy in PKO mice.

Perforin-deficient mice (PKO) and wild-type controls (WT) were infected with 200 pfu LCMV-WE intravenously. On day 5 postinfection (p.i.), mice remained untreated (PKO, WT) or received an adoptive transfer of 1 × 10^7 lymphocytes or 4 × 10^6 purified CD3 T cells from LCMV-immune wild-type mice (PKO + ATCT). Transferred CD8 T cells (trsf.) were distinguished from endogenous CD8 T cells (endog.). Untreated PKO mice were analyzed on day 12 p.i., PKO mice with transferred cells, and WT mice on day 25–30 after therapy.

A–D Endogenous and transferred CD8 T cells were analyzed by flow cytometry: $KLRG1^+$ and/or $CD127^+$ (A), $PD\text{-}1^+LAG3^+$ (B), $TIM3^+TCF\text{-}1^-$ (C) and TOX^+ (D) (n = 9 PKO, 9 PKO + ATCT, 7 WT). Data information: Data are mean ± SEM with n (A–D) 7–9 mice in 3 experiments. Statistics: Mann–Whitney test (A–D). ****$P \leq 0.0001$.

E Exemplary gating strategy: (1) gating on lymphocytes; (2) exclusion of doublets; (3) determination of the frequency of CD4 and CD8 T cells, gating on CD8 T cells; (4) discrimination of $CD45.1^+$ and $CD45.2^+$ CD8 T cells; (5) analysis of $CD45.1^+$ or $CD45.2^+$ LCMV-GP_{33-41}-specific CD8 T cells.

© 2022 The Authors
