# Supplementary material for: Adoptive T cell therapy cures mice from active hemophagocytic lymphohistiocytosis (HLH)
Source: EMBO Mol Med. 2022 Oct 24;14(12):e16085. doi: 10.15252/emmm.202216085 (PMC9728053; doi:10.15252/emmm.202216085)
Supplement: Supplementary file 6 [file EMMM-14-e16085-s006.pdf]

EMBO Molecular Medicine

Adoptive T cell therapy cures mice from active hemophagocytic lymphohistiocytosis (HLH)

Kristoffer Weißert, Sandra Ammann, Tamara Kögl, Viviane Dettmer-Monaco, Christoph Schell, Toni Cathomen, Stephan Ehl, and Peter Aichele
DOI: 10.15252/emmm.202216085

Corresponding author: Peter Aichele (peter.aichele@uniklinik-freiburg.de)

Review Timeline:

Submission Date:	5th Apr 22
Editorial Decision:	12th May 22
Appeal Received:	1st Jun 22
Editorial Decision:	7th Jun 22
Revision Received:	6th Jul 22
Editorial Decision:	26th Jul 22
Revision Received:	30th Aug 22
Editorial Decision:	19th Sep 22
Revision Received:	27th Sep 22
Accepted:	29th Sep 22

Editor: Lise Roth

Transaction Report:

(Note: With the exception of the correction of typographical or spelling errors that could be a source of ambiguity, letters and reports are not edited. Depending on transfer agreements, referee reports obtained elsewhere may or may not be included in this compilation. Referee reports are anonymous unless the Referee chooses to sign their reports.)

12th May 2022

Decision on your manuscript EMM-2022-16085

Dear Dr. Aichele,

Thank you for the submission of your manuscript to EMBO Molecular Medicine, and please accept my apologies for the delay in getting back to you, as one referee needed more time to complete his/her report. We have now received feedback from the two referees who agreed to evaluate your work.
As you will see from the reports below, while referee #2 is overall supportive of publication pending addition of human data, referee #1 is more critical of the study, in particular due to the animal model used that does not reflect the human situation, and to the unclear clinical benefit of the strategy employed.

Given the nature of these concerns and the fact that human relevance is crucial for publication in EMBO Molecular Medicine, I am afraid I see little choice but to return the manuscript to you at this point with the decision that we cannot offer to publish it.

I am very sorry to disappoint you in this occasion, and hope that the referees' comments are helpful in your continued work in this area.

With kind regards,

Lise Roth

Lise Roth, Ph.D
Senior Editor
EMBO Molecular Medicine

***** Reviewer's comments *****

Referee #1 (Comments on Novelty/Model System for Author):

The animal model selected by the Authors does not have practical clinical translation, since the disease is in the mice is triggered by a specific virus and the Authors infused syngeneic antigen-experienced T cells.

Referee #1 (Remarks for Author):

This manuscript does not contain any clinically relevant information. In addition, from a conceptual point of view, the results observed are more than expected and there is nothing new. Indeed, the efficacy of allogeneic HSCT is exactly based on the fact that donor T cells with an intact cytotoxicity machinery are either adoptively transferred with the graft or differentiate from the hematopoietic stem cells of the donor. This, in turn, results into termination of chronic antigen stimulation and of hyperactivation of monocyte/macrophage cells.
The mouse model where the cells infused have been obtained by a syngeneic donor does not encounter any equivalent in the clinical situation.
It is really surprising that the Authors can believe that these third-party, allogeneic, virus-specific T cells can survive enough in a human being and to expand without any profound immune-suppression of the recipient. How do they think that these third-party allogeneic, virus-specific T cells are not eliminated by the patient immune system, which is hyperactivated? Which evidence do the Authors have that after infusion in non-immune-suppressed human beings third-party, allogeneic, virus-specific T cells surive? In the absence of this evidence, the model developed has limited value, if any.
Do the Authors think to create a cell bank of third-party, allogeneic, virus-specific T cell? Is this really competitive with the currently available therapies, including the novel agents ruxolitinib and, at least for the US patients, emapalumab?
A further limitation of the approach, just mentioned by the Authors, but not fully discussed, is that a viral trigger is identified only in a minority of patients. Do the Authors think to infuse third-party, allogeneic, pathogen-specific T cells against the all known pathogens?
The Authors seem to have confused idea also on how gene addition therapy works. Indeed, the cells transduced in gene addition therapy approaches are hematopoietic stem cells and not T lymphocytes. The latter elements were used in pioneering studies more than 20 years ago.
The statement "Clinical management focusses on initial aggressive immunosuppression with etoposide, anti-thymocyte globulin (ATG) or alemtuzumab, cyclosporine A and dexamethasone" is vague. A more elaborated discussion of the currently employed

therapies in primary HLH is highly recommended.

Referee #2 (Remarks for Author):

In current manuscript by Weissert et al. the authors describe a proof-of-concept of adoptive T cell therapy (ATCT) in HLH. Hemophagocytic lymphohistiocytosis is a rare and deadly disease in patients, which is very difficult to treat. I want to congratulate the authors for this comprehensive and important study.

Major point: The authors identify a marker combination in the mouse studies that "protects" most mice from HLH. Did the authors also test their findings in patients with HLH? Is this observation of clinical relevance? The current studies, which is mainly based on animal models, would benefit from additional data in humans.

Minor points:
Introduction: please address the incidence of HLH in humans
Fig. 1C: define scale bars
Result section (page 8). Indicate the relevance of CD107a staining in the text

Subject: rebuttal letter to manuscript EMM-2022-16085

Referee #1 (Comments on Novelty/Model System for Author):

The animal model selected by the Authors does not have practical clinical translation, since the disease is in the mice is triggered by a specific virus and the Authors infused syngeneic antigen-experienced T cells.

We respectfully disagree with the reviewer that the LCMV-Jinx (MUNC13-4 deficiency) and LCMV-PKO (PERFORIN-deficiency) animal models used in this study do not have practical clinical translation for HLH patients.

There is little debate that induction of HLH in humans requires a trigger. Most FHL patients are born asymptomatically and activate in the context of a trigger. In around 20% of primary HLH cases this is an infectious trigger, with EBV and CMV being the main culprits. In the other 80%, no infectious trigger can be identified at the time of diagnosis. It is currently unclear whether infectious or inflammatory agents escape detection or have already been eliminated at the time of diagnosis. In PKO mice, disease can be triggered with LCMV or MCMV, systemic persistent viruses just as EBV and CMV in HLH patients. Infected mice develop all features used for diagnosis of the human disease. Based on these considerations, the HLH-prone mice are excellent models for primary HLH and have been absolutely essential to understand the pathogenesis of this disease. Infusion of syngeneic antigen-experienced T cells is a therapeutic strategy that is used in innova-

tive clinical protocols that use gene-corrected autologous cells to fight cancer, infectious diseases or human genetic diseases. For HLH, such protocols are developed for XLP (https://doi.org/10.1016/j.jaci.2018.02.053), for FHL2 (https://doi.org/10.1016/j.jaci.2017.11.050, ref. 26 in the manuscript) and FHL3 (https://doi.org/10.1089/hum.2019.329, ref. 68 in the manuscript). The concept is to harvest the gene-deficient T cells from individuals undergoing HLH in order to get syngeneic, (trigger-) antigen experienced cells, to correct the genetic defect by gene addition or gene editing and then to re-infuse the cells.

Referee #1 (Remarks for Author):

1. This manuscript does not contain any clinically relevant information.

We respectfully disagree with the reviewer. We consider the preclinical evaluation of an innovative, promising, but potentially dangerous therapy in an accepted murine model for the human disease, for which therapeutic options are still not satisfactory, as clinically relevant.

2. In addition, from a conceptual point of view, the results observed are more than expected and there is nothing new.

We respectfully disagree with the reviewer. It is expected that the equipment of cytotoxicity-deficient mice with a cytotoxicity-competent immune system will protect these mice from developing HLH following a trigger infection. This is what has been done in the gene therapy studies mentioned above. But this is **NOT** what we have done in this study. We have deliberately chosen the clinically relevant situation, where disease had already been triggered and mice presented with acute hyperinflammation. The key question was whether adding competent virus-specific T cells to this hyperinflammatory situation will be safe and effective. Do T cells infused into mice with highly active HLH survive? Can they control the virus and attenuate the ongoing hyperinflammation? Can they do that without killing the mouse? These important questions have far-reaching clinical implications.

3. Indeed, the efficacy of allogeneic HSCT is exactly based on the fact that donor T cells with an intact cytotoxicity machinery are either adoptively transferred with the graft or differentiate from the hematopoietic stem cells of the donor. This, in turn, results into termination of chronic antigen stimulation and of hyperactivation of monocyte/macrophage cells.

With all due respect, the reviewer has misunderstood a very simple fact of this paper. The experiments in our manuscript target phase I of HLH therapy i.e. the initial control of hyperinflammation – BEFORE HSCT is performed. They do not address phase II of HLH therapy, i.e. donor stem cell transfusion including some donor T cells that prevent recurrence of the disease. As a perspective

VST can be used in refractory virus-triggered 1° and 2° HLH to eliminate the trigger, terminate disease progression and allow HSCT.

4. The mouse model where the cells infused have been obtained by a syngeneic donor does not encounter any equivalent in the clinical situation.

We respectfully disagree with the reviewer and refer to the examples given in our response to the first comment. We agree that we should more explicitly explain the clinically targeted situation in our manuscript. The equivalent clinical situation would be the transfer of gene-corrected autologous T cells. This is a proof-of-concept study demonstrating that ATCT is possible under conditions of hyperinflammation in HLH.

5. It is really surprising that the Authors can believe that these third-party, allogeneic, virus-specific T cells can survive enough in a human being and to expand without any profound immune-suppression of the recipient. How do they think that these third-party allogeneic, virus-specific T cells are not eliminated by the patient immune system, which is hyperactivated?

We are surprised about the limited understanding of the HLH treatment phases by the reviewer. HLH patients initially receive substantial cytoreductive immunosuppressive therapy. Immunosuppression of the recipient is therefore clearly expected in our targeted clinical treatment situation, i.e. where control of hyperinflammation cannot be achieved by these measures. In this immunosuppressed situation, gene-corrected autologous T cells are not expected to be eliminated. And there is also evidence from several studies, that successful ATCT based on VST is possible for patients post HSCT (https://doi.org/10.1186/s13045-019-0701-1, Ref. 23 in the manuscript), for patients with inborn errors of immunity (https://doi.org/10.1016/j.jaci.2015.12.1311; https://doi.org/10.1016/j.jaip.2018.10.049; https://doi.org/10.1182/blood.2019000924, ref. 20,21,24 in the manuscript) and for patients with solid organ transplantation (https://doi.org/10.1111/tri.12659; doi.org/10.3389/fimmu.2021.657144). Allogeneic virus-specific T cells persist long enough to convey therapeutic efficacy in transfused hosts (https://doi.org/10.1182/bloodadvances.2017010223; https://doi.org/10.1200/JCO.2017.73.0655; Ref.53, 54 in the manuscript). Especially for VST therapy in the context of inborn errors of immunity or solid organ transplantation, patients are not always severely lymphopenic at the time of transfer.

In our experimental situation, we first wanted to address efficacy and safety in the absence of prior immunosuppression. Again, we take this comment as a motivation to more explicitly explain the targeted clinical situation.

UNIVERSITÄTS KLINIKUM FREIBURG

6. Which evidence do the Authors have that after infusion in non-immune-suppressed human beings third-party, allogeneic, virus-specific T cells surive? In the absence of this evidence, the model developed has limited value, if any.

The main targeted clinical situations in HLH are the transfusion of gene-corrected autologous T cells or the transfer of partially HLA-matched VST in the case of virus-triggered disease. As mentioned above (point 5), studies in patients demonstrated that allogeneic virus-specific T cells persist long enough to convey therapeutic efficacy. VST application was even done without conditioning in some patients with inborn errors of immunity or in pre-HSCT patients (https://doi.org/10.1016/j.jaci.2015.12.1311; https://doi.org/10.1016/j.jaip.2018.10.049; https://doi.org/10.1182/blood.2019000924, Refs. 20, 21, 24 in the manuscript).

Moreover, in order to assure safety and efficacy, translational medicine is a careful step-by-step approach. We decided to do these proof-of-principle experiments, where we worried most about immunopathology caused by the transfused T cells, in the absence of immunosuppression. We considered this the most sensitive read-out to detect detrimental effects that could be caused by the transfused cells.

7. Do the Authors think to create a cell bank of third-party, allogeneic, virus-specific T cell? Is this really competitive with the currently available therapies, including the novel agents ruxolitinib and, at least for the US patients, emapalumab?

The authors mainly think of using partially HLA-matched virus-specific T cells in virus-triggered HLH and, in future, gene-corrected autologous T cells in situations, where current therapies are not able to control the HLH hyperinflammation. Several institutions have already started to establish such cell banks that include virus-specific T cells directed against one or several viruses (https://doi.org/10.3389/fimmu.2012.00410; https://doi.org/10.1200/JCO.2017.73.0655; https://doi.org/10.1126/scitranslmed.3008825; ref. 52, 53, 61 in the manuscript). In addition, there are several ongoing phase II/III clinical trials with virus-specific T cells of partially HLA-matched donors supporting clinical relevance of the therapy (NCT02532452, NCT04832607; NCT03266640; NCT04364178).

Due to the rather discouraging results from emapalumab monotherapy and the need to use additional cytoreductive therapies in many cases, this drug was not licensed in Europe. There are very limited data on ruxolitinib for the primary treatment of hereditary HLH, but the first unofficial results on ruxolitinib as a monotherapy are not encouraging. Therefore, there is a clear clinical need for new treatment approaches.

We agree that this could be stated more explicitly in the manuscript.

8. A further limitation of the approach, just mentioned by the Authors, but not fully discussed, is that a viral trigger is identified only in a minority of patients. Do the Authors think to infuse third-party, allogeneic, pathogen-specific T cells against the all known pathogens?

The advantage of using gene-corrected autologous T cells is that they will contain (trigger-) antigen-specific T cells – whatever the trigger is. It will therefore not be required to identify a trigger when using this approach. In the case of virus-triggered HLH, the disease is associated with manageable number of pathogens (mainly EBV and CMV) which could be targeted with VST.

9. The Authors seem to have confused idea also on how gene addition therapy works. Indeed, the cells transduced in gene addition therapy approaches are hematopoietic stem cells and not T lymphocytes. The latter elements were used in pioneering studies more than 20 years ago.

We are again surprised by the limited view of this reviewer on current concepts of cellular gene therapy, in particular in the field of primary immunodeficiencies such as HLH (https://doi.org/10.1111/bjh.17070). We have mentioned the T cell gene therapy studies in XLP, FHL2, FHL3 above. We would like to add T cell gene therapy of IPEX (https://doi.org/10.1002/eji.202149210) and CD40L (https://doi.org/10.1182/blood-2015-11-683235). Many more studies are ongoing in other fields, particularly in HIV and cancer immunotherapy.

10. The statement "Clinical management focusses on initial aggressive immunosuppression with etoposide, anti-thymocyte globulin (ATG) or alemtuzumab, cyclosporine A and dexamethasone" is vague. A more elaborated discussion of the currently employed therapies in primary HLH is highly recommended.

We thank the reviewer for this suggestion and will address this issue in a revised version.

Referee #2 (Remarks for Author):

In current manuscript by Weissert et al. the authors describe a proof-of-concept of adoptive T cell therapy (ATCT) in HLH. Hemophagocytic lymphohistiocytosis is a rare and deadly disease in patients, which is very difficult to treat. I want to congratulate the authors for this comprehensive and important study.

Major point: The authors identify a marker combination in the mouse studies that "protects" most mice from HLH. Did the authors also test their findings in patients with HLH? Is this observation of

clinical relevance? The current studies, which is mainly based on animal models, would benefit from additional data in humans.

This is an important question, that is, however, difficult to address. The ideal situation would be to compare T cell differentiation in patients with self-limited HLH, in which T cells are eventually able to control the hyperactivation, to T cell differentiation in patients with progressive HLH. Such samples are very difficult to obtain. It would be possible to determine whether T cells from patients with untreated HLH phenotypically resemble T cells from mice that fail to control the disease, but we do not think that these findings can be easily related to the therapeutic murine T cells.

Minor points:

Introduction: please address the incidence of HLH in humans

Fig. 1C: define scale bars

Result section (page 8). Indicate the relevance of CD107a staining in the text

We thank the reviewer for the suggestions and will address these issues in a revised version.

Yours sincerely

Dr. Peter Aichele

7th Jun 2022

Dear Dr. Aichele,

Thank you for your request to appeal our decision on your manuscript.
As mentioned in a previous correspondence, I have read your appeal letter and point-by-point rebuttal and discussed them with my colleagues.
We would like to invite you to submit your revised work together with a point-by-point letter, that we will submit for evaluation to 1 or 2 additional advisors/referees.
Please note that we cannot guarantee at this stage that the eventual outcome will be favorable.

Revised manuscripts should be submitted within three months of a request for revision; they will otherwise be treated as new submissions, except under exceptional circumstances in which a short extension is obtained from the editor.

I look forward to seeing a revised form of your manuscript as soon as possible. Please use this link to login to the manuscript system and submit your revision:

Yours sincerely,

Lise Roth

Lise Roth, Ph.D
Senior Editor
EMBO Molecular Medicine

Referee #1 (Comments on Novelty/Model System for Author):

The animal model selected by the Authors does not have practical clinical translation, since the disease is in the mice is triggered by a specific virus and the Authors infused syngeneic antigen-experienced T cells.

We respectfully disagree with the reviewer that the LCMV-Jinx (MUNC13-4 deficiency) and LCMV-PKO (PERFORIN-deficiency) animal models used in this study do not have practical clinical translation for HLH patients. Induction of HLH in humans very likely requires a trigger. Most FHL patients are born asymptomatically and activate at different time points, mostly in the first year of life. In around 20% of primary HLH cases an infectious trigger can be identified at the time of disease activation, with EBV and CMV being the main culprits. In the other 80%, no infectious trigger can be detected at the time of diagnosis. It is currently unclear whether infectious or inflammatory agents escape detection or have already been eliminated at the time of diagnosis *(see p3, line 56-61)*. In PKO mice, disease can be triggered with LCMV or MCMV, systemic persistent viruses just as EBV and CMV in HLH patients. Infected mice develop all features used for diagnosis of the human disease *(see p5, line 95-100)*. Based on these considerations, the HLH-prone mice are excellent models for primary HLH and have been absolutely essential to understand the pathogenesis of this disease and to test therapeutic interventions (anti-IFNγ antibody treatment / JAK1/2 inhibition).

Infusion of syngeneic antigen-experienced T cells is a therapeutic strategy that is used in innovative clinical protocols that utilize gene-corrected autologous cells to fight cancer, infectious diseases or human genetic diseases (https://doi.org/10.1016/j.cell.2020.03.001, ref. “Weber *et al*, 2020” in the manuscript). For HLH, such protocols are developed for XLP (https://doi.org/10.1016/j.jaci.2018.02.053, ref. “Panchal *et al*, 2018” in the manuscript), for FHL2

(https://doi.org/10.1016/j.jaci.2017.11.050, ref. "Ghosh *et al*, 2018" in the manuscript) and FHL3 (https://doi.org/10.1089/hum.2019.329, ref. "Takushi *et al*, 2020" in the manuscript). The concept is to harvest the gene-deficient T cells from individuals undergoing HLH in order to get syngeneic, trigger-antigen-experienced T cells, to correct the genetic defect by gene addition or gene editing and then to re-infuse the cells *(see p4, line 83-92)*.

Referee #1 (Remarks for Author):

1. This manuscript does not contain any clinically relevant information.

We respectfully disagree with the reviewer. We consider the preclinical evaluation of an innovative, promising, but potentially dangerous therapy in an accepted murine model for the human disease, for which therapeutic options are still not satisfactory, as clinically relevant.

2. In addition, from a conceptual point of view, the results observed are more than expected and there is nothing new.

We respectfully disagree with the reviewer. It is expected that the equipment of cytotoxicity-deficient mice with a cytotoxicity-competent immune system will protect these mice from developing HLH following a trigger. This is what has been done in the gene therapy studies mentioned above *(see p4, line 88-92)*. But this is **NOT** what we have done in this study. We have deliberately chosen the clinically relevant situation, where disease had already been triggered and mice presented with acute hyperinflammation. The key question was whether adding competent trigger-specific T cells to this hyperinflammatory situation will be safe and effective. Do T cells infused into mice with highly active HLH survive? Can they control the virus and attenuate the ongoing hyperinflammation? Can they do that without killing the mouse? These important questions have far-reaching clinical implications *(see p5, line 106-113)*.

3. Indeed, the efficacy of allogeneic HSCT is exactly based on the fact that donor T cells with an intact cytotoxicity machinery are either adoptively transferred with the graft or differentiate from the hematopoietic stem cells of the donor. This, in turn, results into termination of chronic antigen stimulation and of hyperactivation of monocyte/macrophage cells.

With all due respect, the reviewer has misunderstood a very simple fact of this paper. The experiments in our manuscript target phase I of HLH therapy i.e. the initial control of hyperinflammation – BEFORE HSCT is performed *(see p4, line 79-88)*. They do not address phase II of HLH therapy, i.e. donor stem cell transfusion including some donor T cells that prevent recurrence of the disease.

4. The mouse model where the cells infused have been obtained by a syngeneic donor does not encounter any equivalent in the clinical situation.

We respectfully disagree with the reviewer and refer to the examples given in our response to the first comment. We agree that we should more explicitly explain the clinically targeted situation in our manuscript. The equivalent clinical situation would be the transfer of gene-corrected autologous T cells. This is a proof-of-concept study demonstrating that ATCT is possible under conditions of hyperinflammation in HLH *(see p4, line 79-88 and p5 line 106-114)*.

5. It is really surprising that the Authors can believe that these third-party, allogeneic, virus-specific T cells can survive enough in a human being and to expand without any profound immune-suppression of the recipient. How do they think that these third-party allogeneic, virus-specific T cells are not eliminated by the patient immune system, which is hyperactivated?

We are surprised about the limited understanding of the HLH treatment phases by the reviewer. HLH patients initially receive substantial cytoreductive immunosuppressive therapy. Immunosuppression of the recipient is therefore clearly expected in our targeted clinical treatment situation, i.e. where control of hyperinflammation cannot be achieved by these measures. In this immunosuppressed situation, transferred T cells are not expected to be eliminated *(see p15/16, line 337-340 and 360-367)*. In our experimental situation, we first wanted to address efficacy and safety in the absence of prior immunosuppression. Again, we take this comment as a motivation and have more explicitly explained the targeted clinical situation.

6. Which evidence do the Authors have that after infusion in non-immune-suppressed human beings third-party, allogeneic, virus-specific T cells surive? In the absence of this evidence, the model developed has limited value, if any.

The main targeted clinical situations in HLH are the transfusion of gene-corrected autologous T cells. A possible use of allogeneic VST is discussed on p14/15 line 327-347. Moreover, in order to assure safety and efficacy, translational medicine is a careful step-by-step approach. We decided to do these proof-of-concept experiments, where we worried most about immunopathology caused by the transfused T cells, in the absence of immunosuppression. We considered this the most sensitive read-out to detect detrimental effects under conditions of hyperinflammation that could be caused by the transfused cells.

7. Do the Authors think to create a cell bank of third-party, allogeneic, virus-specific T cell? Is this really competitive with the currently available therapies, including the novel agents ruxolitinib and, at least for the US patients, emapalumab?

The authors mainly think of using gene-corrected autologous T cells in situations, where current therapies are not able to control the HLH hyperinflammation.

For VST, several institutions have already started to establish such cell banks that include T cells directed against one or several *(see p15, line 342-347)*. In addition, there are several ongoing phase II/III clinical trials with virus-specific T cells of partially HLA-matched donors supporting clinical relevance of the therapy (NCT02532452, NCT04832607; NCT03266640; NCT04364178).

Due to the rather discouraging results from emapalumab monotherapy and the need to use additional cytoreductive therapies in many cases, this drug was not licensed in Europe. There are very limited data on ruxolitinib for the primary treatment of hereditary HLH, but the first unofficial results on ruxolitinib as a monotherapy are not encouraging. Therefore, there is a clear clinical need for new treatment approaches. We have stated this more explicitly in the manuscript (*see p4, line 72-78*).

8. A further limitation of the approach, just mentioned by the Authors, but not fully discussed, is that a viral trigger is identified only in a minority of patients. Do the Authors think to infuse third-party, allogeneic, pathogen-specific T cells against the all known pathogens?
The advantage of using gene-corrected autologous T cells is that they will contain trigger-antigen-experienced T cells – whatever the trigger is (*see p14, line 309-311*). It will therefore not be required to identify a trigger when using this approach.

9. The Authors seem to have confused idea also on how gene addition therapy works. Indeed, the cells transduced in gene addition therapy approaches are hematopoietic stem cells and not T lymphocytes. The latter elements were used in pioneering studies more than 20 years ago.
We are again surprised by the limited view of the reviewer on current concepts of cellular gene therapy, in particular in the field of primary immunodeficiencies such as HLH (https://doi.org/10.1111/bjh.17070; ref. “Panchal *et al*, 2018“ in the manuscript). We have mentioned the T cell gene therapy studies in XLP, FHL2, FHL3 above. We would like to add T cell gene therapy of IPEX (https://doi.org/10.1002/eji.202149210; ref. “Borna *et al*, 2022” in the manuscript) and CD40L (https://doi.org/10.1182/blood-2015-11-683235; ref. “Hubbard *et al*, 2016” in the manuscript). Many more studies are ongoing in other fields, particularly in HIV and cancer immunotherapy (*see p4 line 88-90 and p14 line 322-326*).

10. The statement "Clinical management focusses on initial aggressive immunosuppression with etoposide, anti-thymocyte globulin (ATG) or alemtuzumab, cyclosporine A and dexamethasone"

is vague. A more elaborated discussion of the currently employed therapies in primary HLH is highly recommended.

We thank the reviewer for this suggestion and will address this issue in the revised version (*see p3/4 line 62-78*).

Referee #2 (Remarks for Author):

In current manuscript by Weissert et al. the authors describe a proof-of-concept of adoptive T cell therapy (ATCT) in HLH. Hemophagocytic lymphohistiocytosis is a rare and deadly disease in patients, which is very difficult to treat. I want to congratulate the authors for this comprehensive and important study.

We thank the reviewer for the positive comments on our manuscript.

Major point: The authors identify a marker combination in the mouse studies that "protects" most mice from HLH. Did the authors also test their findings in patients with HLH? Is this observation of clinical relevance? The current studies, which is mainly based on animal models, would benefit from additional data in humans.

This is an important question, that is, however, difficult to address. The ideal situation would be to compare T cell differentiation in patients with self-limited HLH, in which T cells are eventually able to control the hyperactivation, to T cell differentiation in patients with progressive HLH. Such samples are very difficult to obtain. It would be possible to determine whether T cells from patients with untreated HLH phenotypically resemble T cells from mice that fail to control the disease, but we do not think that these findings can be easily related to the therapeutic murine T cells.

Minor points:

Introduction: please address the incidence of HLH in humans

We have included the incidence of HLH (*see p3, line 47-50).*

Fig. 1C: define scale bars

Please see legend Figure 1 (see *p35, line 789*).

Result section (page 8). Indicate the relevance of CD107a staining in the text

CD107a is located on lytic granules. Surface expression of CD107a is only detectable if the T cells degranulate and release the content. Reduced CD107a surface expression is a marker for

UNIVERSITÄTS KLINIKUM FREIBURG

reduced degranulation capacity of the T cells leading to reduced cytolytic activity *(see p6, line 130-132).*

Description of changes in revision 2 of EMM-2022-16085

Implemented all required revisions by the journal staff:

1. Changed all Figure legends of the manuscript according to the required standard of EMBO Mol Med.
2. Included "Data and software availability" section to Material and Methods.
3. Used CRediT nomenclature for author contributions.
4. Renamed the corresponding section "Disclosure Statement and Competing Interests".
5. Added a "The Paper Explained" section to the manuscript.
6. Adjusted references to EMBO style.
7. Deleted the respective sentence including "data not shown": ("Transfer of memory CD4 T cells alone was not curative in Jinx mice (data not shown)".
8. Replaced the callouts for Figure 7D and Figure 9 in the manuscript with the correct figure references.

In addition, statistics of the following figures were re-calculated (in the respective cases, unpaired t-test was shown instead of the necessary Mann-Whitney test):

- Fig. 2E * instead of ** comparing Jinx+ATCT vs. WT
- Fig. 2F * instead of ns comparing Jinx+ATCT vs. Jinx
- Fig. 2K * Jinx vs. Jinx+ATCT, ns Jinx+ATCT vs. WT, * Jinx vs WT
- Fig. 5 inserted statistics that were lost due to a copy-paste mistake

➔Consequently, the respective figures and text passages have been adapted (Figure 2, 4D, 5)

The population "PD-1/LAG3^{-}" was renamed to "PD-1^{low}LAG3^{low}", as the authors agreed that this description is appropriate when describing the downregulation of inhibitory receptors PD-1 and LAG3 (compare Figure 3B, column I).

- Adapted Fig. 6, Fig. 7 and Figure EV4 accordingly
- Adapted the text passages in the manuscript
  - page 11, line 247
  - page 38, line 861-865
  - page 39, line 878
  - page 39, line 893
  - page 43, line 952-959

Furthermore, the following changes to the manuscript have been done according to the guidelines of EMBO Mol Med for revised manuscripts:

- Inserted Keywords after Abstract
- Inserted antibody list in Material and Methods section
- Checklist excel-table was changed
  - reference to antibody list was added.
  - added information, that mouse experiments were conducted according to the ARRIVE guidelines.

- Replaced Supplementary information with Expanded View and added Expanded View Figure legends to the manuscript
- References were updated in the point to point draft according to EMBO style

26th Jul 2022

Dear Dr. Aichele,

Thank you for resubmitting your manuscript to EMBO Molecular Medicine. We have now received feedback from the two new reviewers who agreed to evaluate your manuscript (as well as the point-by-point letter). As you will see from the reports below, while referee #3 is overall supportive of publication pending minor revisions, referee #4 remains unconvinced by the clinical relevance of the findings.

Given that clinical/translational relevance is a key criterion for consideration in EMBO Molecular Medicine, we have discussed this issue further with the referees.
They both agreed that further proof of concept should be provided, either by showing that T cell subsets from patients resemble those observed in your preclinical models, or by inducing HLH in Jinx mice with LCMV infection, taking T cells during the active phase, retrovirally transducing them with the corrected gene (UNC or PRF), and reinfusing them into syngeneic Jinx recipients with newly induced active HLH induced by LCMV.
On this point, referee #4 further commented:
"I think their data argues this may not work since LCMV-specific effector T cells from wild type mice were ineffective, however the results would be important whether positive or negative, and if effective, this would be a critical proof of concept.
This experiment, although difficult is feasible and would be ideal."

Furthermore, a clear discussion on the clinical relevance of your findings should be provided in the manuscript text.

If you feel you can satisfactorily address these points and those listed by the referees, you may wish to submit a revised version of your manuscript. If you would like to discuss the revisions further, please feel free to contact me. When resubmitting your manuscript, kindly attach a covering letter giving details of the way in which you have handled each of the points raised by the referees. A revised manuscript will once again be subject to review.

Please also address the following editorial issues:
- Keywords: Please provide up to 5 keywords
- Author Contributions: please remove from the manuscript; CRediT has replaced the traditional author contributions section because it offers a systematic machine readable author contributions format that allows for more effective research assessment. You are encouraged to use the free text boxes beneath each contributing author's name to add specific details on the author's contribution. More information is available in our guide to authors.
- Source Data: We would also encourage you to include the source data for figure panels that show essential data. Numerical data should be provided as individual .xls or .csv files (including a tab describing the data). For blots or microscopy, uncropped images should be submitted (using a zip archive if multiple images need to be supplied for one panel). Additional information on source data and instruction on how to label the files are available at
.
- Synopsis: Every published paper now includes a 'Synopsis' to further enhance discoverability. Synopses are displayed on the journal webpage and are freely accessible to all readers. They include a short stand first (maximum of 300 characters, including space) as well as 2-5 one-sentences bullet points that summarizes the paper. Please write the bullet points to summarize the key NEW findings. They should be designed to be complementary to the abstract - i.e. not repeat the same text. We encourage inclusion of key acronyms and quantitative information (maximum of 30 words / bullet point). Please use the passive voice. Please attach these in a separate file or send them by email, we will incorporate them accordingly.
Please also suggest a striking image or visual abstract to illustrate your article as a PNG/TIFF/JPEG file 550 px wide x 300-600 px high.
- Review Process File: As part of the EMBO Publications transparent editorial process initiative, EMBO Molecular Medicine will publish online a Review Process File (RPF) to accompany accepted manuscripts.
In the event of acceptance, this file will be published in conjunction with your paper and will include the anonymous referee reports, your point-by-point response and all pertinent correspondence relating to the manuscript. Let us know whether you agree with the publication of the RPF and as here, if you want to remove or not any figures from it prior to publication.
Please note that the Authors checklist will be published at the end of the RPF.

I look forward to receiving your revised manuscript.

Yours sincerely,

Lise Roth

Lise Roth, PhD

Senior Editor
EMBO Molecular Medicine

***** Reviewer's comments *****

Referee #3 (Comments on Novelty/Model System for Author):

The authors provide convincing data on the effectiveness of adoptive T cell transfer for the treatment of hemophagocytic lymphohistiocytosis (HLH) in two mouse models. An important advance is the authors` demonstration that adoptive transfer is possible under hyperinflammatory conditions in HLH. These findings may pave the way to use of gene-corrected autologous T cells in patients with HLH. The main potential limitation is how closely T cells subsets in preclinical models resemble those in patients. This issue has already been raised by Reviewer #2. In my opinion, the manuscript would benefit from addressing this issue (at least in the discussion). I also noted some minor issues that should be addressed to increase the quality of the presentation.

Referee #3 (Remarks for Author):

In the revised version, Weissert et al. provide proof-of-concept of adoptive T cell therapy for the treatment of hemophagocytic lymphohistiocytosis (HLH) in two mouse models. Overall, the rationale and data are presented well, the experiments are properly controlled and well powered, and the conclusions are appropriate. Some specific points may, however, require further attention.

Major Point.
1. The main concern is whether T cell phenotypes observed in the preclinical models resemble those of T cells from patients with untreated HLH. This issue has already been raised by Reviewer #2 during a previous round of review. While obtaining such patient samples can be difficult and beyond the scope of the authors` current study, the manuscript would benefit from addressing this issue along the lines presented in the authors` rebuttal.

Minor Points.

1. A large number of references is incomplete (e.g. Aubert et al 2011; Gather et al, 2020; Haque et al 2001; Hartz et al, 2016; Henter et al, 1991; Hubbard et al, 2016; Jordan et al, 2004 ...).
2. Please remove "Preprint" from those articles published several years before.
3. Line 632. Please check spelling of "L??ngler".
4. Fig. 3 and Fig.EV5. "endog" and "trsf" should be explained in the figure legend.
5. Lines 197-198 should be rephrased.

Referee #4 (Comments on Novelty/Model System for Author):

The work addresses treatment of acute HLH induced by LCMV in a mouse model with T cells from an LCMV immune wild type donor. The experiments are generally well performed and the findings have some novelty -- specifically that this can be accomplished in this model without worsening the hyperinflammatory state. The medical impact is quite overstated for the reasons outlined in the critique and the relevance of the model -- given differences in viral trigger -- is unclear.

Referee #4 (Remarks for Author):

The manuscript by Weißert et al evaluates the use of adoptive T cell therapy with syngeneic lymphocytes to treat active hemophagocytic lymphohistiocytosis (HLH) induced by lymphocytic choriomeningitis virus in mouse models of familial HLH. Prior work has shown that prophylactic infusion of syngeneic lymphocytes from wild type mice can prevent HLH induction in mice. The key new point of the paper is that infusing LCMV-specific memory T cells during the acute hyperinflammatory state of HLH can clear LCMV and resolve HLH without provoking more serious inflammation. Notably, naïve T cells and LCMV-specific effector T cells are not effective in resolving HLH. The experiments in the paper are presented clearly, the major issues lie with the clinical relevance of the findings.

Specific comments:

1. The finding that administering LCMV-specific T cells controls LCMV infection is not surprising. The important finding here is that in the setting of acute HLH induced by LCMV, viral control by ACT can be accomplished safely. However, mice are often more tolerant to cytokine storms than humans and it is possible that this approach may still promote toxicity in patients where timing of administration and the nature of the viral trigger are likely to be very different than in the models used here. This should be acknowledged in the discussion.

2. Given the caveat that the mouse is only a model, the authors argue that the data provides support for the potential use of adoptive T cell transfer for the acute phase of HLH in patients. One approach they suggest is to obtain the patient's autologous T cells during acute HLH, gene correct them, and reinfuse them. The idea is that "trigger"-specific T cells would be present in the host and gene correction would restore the necessary cytotoxic function. Notwithstanding the time constraints in identifying the specific mutation in a patient and performing gene correction of T cells obtained during active HLH, their own data argues that this approach is likely to be ineffective since theey show that only LCMV-specific memory T cells and not LCMV-specific effector T cells had therapeutic efficacy.

3. The key experiment to support gene correcting autologous cells present during acute HLH as a viable approach is feasible and should be performed to support their argument. This would require inducing HLH in Jinx mice, taking T cells during the active phase that would include trigger (LCMV)-specific T cells, retrovirally transducing them with the corrected gene, and reinfusing them into syngeneic jinx recipients with active HLH. Lacking this experiment, the clinical relevance is debatable.

4. The alternative approach the authors suggest for translating their findings to the clinic would be to infuse 3rd party virus-specific T cells. Here again there are very significant limitations. Third party VSTs only occasionally survive long term, even in highly immunosuppressed patients where the efficacy is far from uniform and the mechanism of activity not fully understood. The consequences of an alloreactive host response to the infused 3rd party cells and how concurrent therapy might affect their function are important unknowns. Finally, as acknowledged by the authors, a viral trigger is not often identified hence the specificity of 3rd party cells would not be known.

5. The dose titration of transferred T cells suggests there is a threshold that must be reached in this model for efficacy. This is likely to depend to some extent on viral load and on the frequency of LCMV-specific memory T cells in the syngeneic T cell inoculum. The phenotyping data is consistent with viral clearance and does not seem to add much. Is there a difference in T cell phenotype after clearance of LCMV in WT mice versus the jinx mice that received ACT?

6. Are both CD8 and CD4 T cells required for efficacy or can CD8 T cells alone be effective?

Referee #3 (Comments on Novelty/Model System for Author):
The authors provide convincing data on the effectiveness of adoptive T cell transfer for the treatment of hemophagocytic lymphohistiocytosis (HLH) in two mouse models. An important advance is the authors` demonstration that adoptive transfer is possible under hyperinflammatory conditions in HLH. These findings may pave the way to use of gene-corrected autologous T cells in patients with HLH. The main potential limitation is how closely T cells subsets in preclinical models resemble those in patients. This issue has already been raised by Reviewer #2. In my opinion, the manuscript would benefit from addressing this issue (at least in the discussion). I also noted some minor issues that should be addressed to increase the quality of the presentation.

We thank the reviewer for the positive judgement of our manuscript.

Referee #3 (Remarks for Author):

Major Point.
1. The main concern is whether T cell phenotypes observed in the preclinical models resemble those of T cells from patients with untreated HLH. This issue has already been raised by Reviewer #2 during a previous round of review. While obtaining such patient samples can be difficult and beyond the scope of the authors` current study, the manuscript would benefit from addressing this issue along the lines presented in the authors` rebuttal.

Answer to major point 1:
We have addressed this issue and inserted a comparison of primary HLH patient data with HLH mouse model data in Figure EV1A-F, inserted an additional subheading "CD8 T cell phenotype in HLH mouse models and FHL patients" in the "Results" section on page 6, line 141-157 and in the "Discussion" section on p18, line 426-434). Table 1 was added to the manuscript including more information regarding the patient material and more information was provided in the "Material and Methods" section (see p20/22, line 470-475, line 500-503).
Since 1°HLH is a very rare disease, availability and access to patient material is limited. We reanalysed 1°HLH patients from a cohort that has been published before by our group (Ammann et al. DOI: 10.1002/eji.201646686). We chose eight FHL-2 patients, seven FHL-3 patients and two FHL-5 patients from the cohort. All patients fulfilled at least 5 out of 8 clinical HLH parameters, were less than one year old and were either untreated or close to therapy start (Table 1). Of note, patients usually develop HLH early during childhood, they are severely ill and often develop marked lymphopenia. Therefore, patient material is only available in small quantities and retained

samples are mostly reserved for diagnostic purposes. For comparison to the human data, we collected data from *Perforin*-deficient (FHL-2) and *Jinx* (FHL-3) mice on day 12 or 15 post infection, respectively. As controls, we included non-infected mice and healthy donors (HD). Based on expression of KLRG-1 (not published in the previous paper) and CD127, we detected CD8 T cells that strongly downregulated CD127 and KLRG1 in patients and mice with acute HLH. In contrast, these cells were only found in very low percentages in non-infected mice and HD. Additionally, a high frequency of CD8 T cells in patients and mice expressed inhibitory receptor PD-1, which is not observed on T cells of non-infected mice and HD. Furthermore, the majority of CD8 T cells with an effector memory phenotype were detectable in mice ($CD44^{+}/CD62L^{-}$) and patients ($CD45RA^{-}/CCR7^{-}$) with 1°HLH. Thus, the phenotype of activated T cells observed in preclinical mouse models resemble those of patients with untreated HLH. This is an important finding, since combinations of these markers described in our manuscript to identify "functional" T cells that predict therapy success in the animal model can be transferred directly to HLH patients. The data are now included as Figure EV1 and described and discussed in the manuscript *(see Results p7 line141-157 and Discussion p18, line 424-434)*. Recent publications of other groups (doi.org/10.1007/s10875-021-01109-0 and doi.org/10.1016/j.jaci.2022.02.028) showing increased LAG3 or TIM3 expression levels on PD-1 positive CD8 T cells in 1° HLH patients, support our findings concerning the HLH-associated T cell phenotypes in human and mice.

Minor Points.

1. A large number of references is incomplete (e.g. Aubert et al 2011; Gather et al, 2020; Haque et al 2001; Hartz et al, 2016; Henter et al, 1991; Hubbard et al, 2016; Jordan et al, 2004 ...).

We have corrected the references – thank you for the detailed analysis of the manuscript.

2. Please remove "Preprint" from those articles published several years before.

We updated all respective references, thereby removing "Preprint".

3. Line 632. Please check spelling of "L??ngler".

Name was corrected (see p30, line 682).

4. Fig. 3 and Fig.EV5. "endog" and "trsf" should be explained in the figure legend.

The following sentence is now included in the figure legends: "Transferred CD8 T cells (trsf.) were distinguished from endogenous T cells (endog.)" (see line 877-878, 1039-1040, 1087-1088).

5. Lines 197-198 should be rephrased.

We have rephrased the sentence *(see p9, line 214-217)*.

Referee #4 (Comments on Novelty/Model System for Author):
The work addresses treatment of acute HLH induced by LCMV in a mouse model with T cells from an LCMV immune wild type donor. The experiments are generally well performed and the findings have some novelty -- specifically that this can be accomplished in this model without worsening the hyperinflammatory state. The medical impact is quite overstated for the reasons outlined in the critique and the relevance of the model -- given differences in viral trigger -- is unclear.

Referee #4 (Remarks for Author):
The manuscript by Weißert et al evaluates the use of adoptive T cell therapy with syngeneic lymphocytes to treat active hemophagocytic lymphohistiocytosis (HLH) induced by lymphocytic choriomeningitis virus in mouse models of familial HLH. Prior work has shown that prophylactic infusion of syngeneic lymphocytes from wild type mice can prevent HLH induction in mice. The key new point of the paper is that infusing LCMV-specific memory T cells during the acute hyperinflammatory state of HLH can clear LCMV and resolve HLH without provoking more serious inflammation. Notably, naïve T cells and LCMV-specific effector T cells are not effective in resolving HLH. The experiments in the paper are presented clearly, the major issues lie with the clinical relevance of the findings.

We thank the reviewer for the positive judgement of our manuscript but also for his critical remarks to improve our manuscript.

Specific comments:
1. The finding that administering LCMV-specific T cells controls LCMV infection is not surprising. The important finding here is that in the setting of acute HLH induced by LCMV, viral control by ACT can be accomplished safely. However, mice are often more tolerant to cytokine storms than humans and it is possible that this approach may still promote toxicity in patients where timing of administration and the nature of the viral trigger are likely to be very different than in the models used here. This should be acknowledged in the discussion.
Answer to comment 1:
This study is a proof-of-concept that ATCT is possible under conditions of hyperinflammation. We chose an experimental set-up without accompanying immunosuppression to be as sensitive as possible to detect toxic side effects of the treatment. In the mouse models the transferred T cells did not aggravate HLH by further fuelling the cytokine storm. These data are very promising. However, we fully agree with the reviewer that we can not rule out detrimental side effects of such a therapeutic approach in patients. A possible strategy for a first application in HLH patients

would be to perform an ATCT therapy under partial immunosuppression that does not interfere with the activity of therapeutic T cells, but lowers the risk of a cytokine storm. Such a statement is now included in the discussion *(see p16, line 382-392)*.

2. Given the caveat that the mouse is only a model, the authors argue that the data provides support for the potential use of adoptive T cell transfer for the acute phase of HLH in patients. One approach they suggest is to obtain the patient's autologous T cells during acute HLH, gene correct them, and reinfuse them. The idea is that "trigger"-specific T cells would be present in the host and gene correction would restore the necessary cytotoxic function. Notwithstanding the time constraints in identifying the specific mutation in a patient and performing gene correction of T cells obtained during active HLH, their own data argues that this approach is likely to be ineffective since they show that only LCMV-specific memory T cells and not LCMV-specific effector T cells had therapeutic efficacy.

Answer to comment 2:

We agree with the reviewer that time lines for gene correction of T cells might be a critical issue, but the techniques for gene correction will improve in the future and may allow such an ambitious approach.

3. The key experiment to support gene correcting autologous cells present during acute HLH as a viable approach is feasible and should be performed to support their argument. This would require inducing HLH in Jinx mice, taking T cells during the active phase that would include trigger (LCMV)-specific T cells, retrovirally transducing them with the corrected gene, and reinfusing them into syngeneic jinx recipients with active HLH. Lacking this experiment, the clinical relevance is debatable.

Answer to comments 2 and 3:

We fully agree with the reviewer that the mouse is a model for 1°HLH in humans. We are convinced that the HLH mice and the development of the disease is very well studied and therefore reflects the best option to evaluate innovative and promising new therapies, as it was done for IFNγ neutralizing antibodies and JAK1/2 inhibition. For ATCT an evaluation in an animal model is mandatory as this therapeutic approach has a potential risk. In a first step, we demonstrated that in principle ATCT in HLH is possible (this manuscript). In further research projects, we want to stepwise adjust the animal model to the clinical situation. Our final goal will be the transfer of gene-corrected autologous T cells. The key experiment suggested by the reviewer is a complex, long-term project with many open questions, which need to be addressed (see p14, line 337-343):

- Is it possible to harvest enough peripheral T cells during active HLH under conditions of hyperinflammation and lymphopenia? This will not be a problem in the mouse model as we can remove spleen and lymph nodes from a diseased *Jinx* donor mouse.
- Is gene correction in hyperactivated T cells possible? We plan to follow a gene editing strategy based on a CRISPR-Cas9 in *Jinx* CD8 T cells. Preliminary experiments indicate that in principle nucleofection of hyperactivated T cells with GFP-coding RNA is possible *(see Figure R1 for the reviewer on page 8 of this letter)*.
- How efficient is gene correction in hyperactivated CD8 T cells? We will use different protocols and editing strategies to optimize gene correction. Off-target activities need to be analysed for the different experimental setups.
- Will hyperactivated gene-corrected T cells survive after transfer? We will test this in non-infected recipients and in recipient mice with HLH under hyperinflammatory conditions. This topic may be the major challenge, as ATCT with effector T cells had no therapeutic effect in our hands. We will elucidate whether enrichment of T cell subpopulations with high proliferative capacity or *in vitro* re-differentiation of gene-corrected T cells will be strategies to solve the problem.

This will be our future experimental strategy for the ATCT project. Currently we are writing proposals to get an approval of the authorities (Regierungspräsidium Freiburg, Germany) for the animal experiments. However, it will most probably take several months to receive the respective permissions, after which pilot experiments can be started.

4. The alternative approach the authors suggest for translating their findings to the clinic would be to infuse 3rd party virus-specific T cells. Here again there are very significant limitations. Third party VSTs only occasionally survive long term, even in highly immunosuppressed patients where the efficacy is far from uniform and the mechanism of activity not fully understood. The consequences of an alloreactive host response to the infused 3rd party cells and how concurrent therapy might affect their function are important unknowns. Finally, as acknowledged by the authors, a viral trigger is not often identified hence the specificity of 3rd party cells would not be known.

Answer to comment 4:

The reviewer is right that the VST approach may have limitations. However, we see a future possibility to intervene in verified virus-triggered HLH with VSTs. We have tried to carefully discuss this issue and to integrate the concerns of the reviewer *(see p15/16, line 354-372)*.

5. The dose titration of transferred T cells suggests there is a threshold that must be reached in this model for efficacy. This is likely to depend to some extent on viral load and on the frequency of LCMV-specific memory T cells in the syngeneic T cell inoculum. The phenotyping data is consistent with viral clearance and does not seem to add much. Is there a difference in T cell phenotype after clearance of LCMV in WT mice versus the jinx mice that received ACT?

Answer to comment 5:

A critical number of LCMV-specific memory T cells in the inoculum is needed to achieve an expansion after transfer that is sufficient to remove the disease trigger and cure the *Jinx* mice from HLH. The T cell inoculum is free of virus, since the LCMV-immune donor mice have completely cleared the virus. The T cell phenotyping was performed early (day 20 after therapy start) and late (more than day 100 after therapy start) after viral clearance. The phenotyping early after viral clearance reveals that T cells in *Jinx* mice with ATCT have a characteristic, intermediate phenotype compared to *Jinx* without ATCT and WT mice (see Figure 3, Figure EV3 and page 8/9, line 193-195, 203-213). Later than day 100 after therapy start, the T cell phenotype after LCMV clearance in WT mice are comparable to the transferred and endogenous CD8 T cells of cured *Jinx* in terms of KLRG1 and/or CD127 expression, PD-1/LAG-3 expression and TIM3 expression *(see p9, line 217-221 and Figure 3A-C, column IV)*.

However, we added now a sentence to the manuscript to stress, that regarding TCF-1 and TOX expression, not all CD8 T cells from cured *Jinx* mice normalized to WT mice *(see p9/10, line 221-223 and Figure EV3A and B, column IV)*. This phenomenon is also seen in HCV infections in humans and is described as a molecular scar of the specific CD8 T cells even after resolution of chronic antigen stimulation (https://doi.org/10.1038/s41590-020-00817-w).

6. Are both CD8 and CD4 T cells required for efficacy or can CD8 T cells alone be effective?

Answer to comment 6:

This is an important point the reviewer addresses. Yes, CD8 T cells alone are sufficient to cure *Jinx* mice from active HLH *(see p11, line 246-251 and Figure 5A)*. Nevertheless, we decided to transfer CD3 T cells in the vast majority of experiments, as CD4 T cells provide critical help to CD8 T cells during chronic infections (see p7, line 163-167).

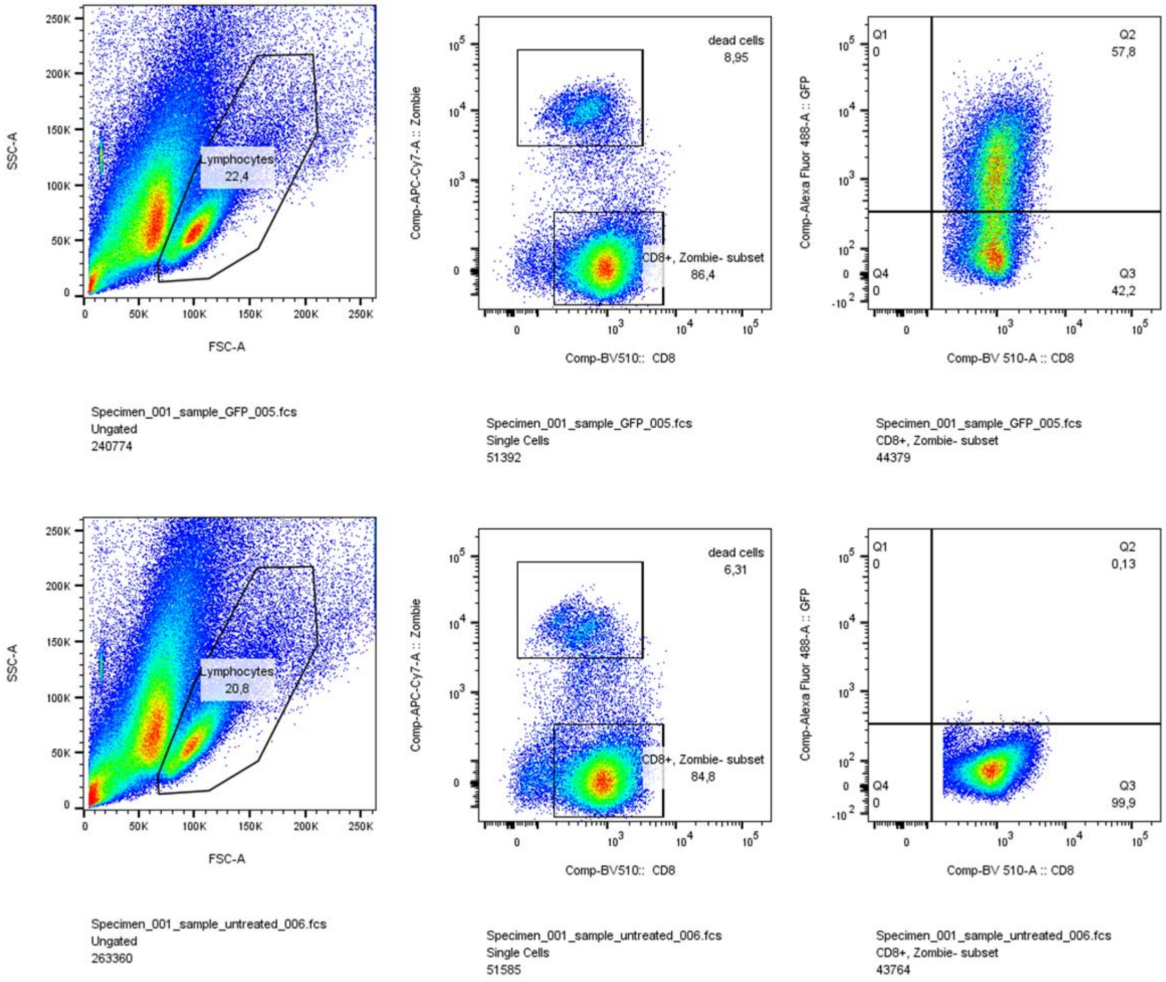

Figure R1. Nucleofection of hyperactivated CD8 T cells. PKO mice were infected with 200 pfu LCMV-WE i.v.. On day 12 p.i., CD8 T cells from spleen were isolated, purified and electroporated using a 4D-Nucleofector Device (Lonza, Switzerland) (upper row) or were not electroporated (lower row). After incubation for 18h in a 37°C incubator, the frequency of living CD8 T cells expressing GFP was determined by flow cytometry.

Description of additional changes in revision 3 of EMM-2022-16085

- Added the following sentence on page 15, line 356-358 in response to reviewer 4, comment 4:
  "Further studies will be necessary to evaluate VST rejection, long-term survival and compatibility with concurrent therapies in patients."
- Moved the following sentence to page 15, line 363-366:
  "Studies in HSCT patients with gene-marked therapeutic T cells or analysis by TCR deep-sequencing demonstrated, that expansion capacity of CD4 and CD8 T cell clonotypes, TCR diversity and persistence of donor T cells correlate with therapeutic efficacy of ATCT (Keller *et al*, 2019)."
- Replaced on page 15, line 370 "could" with "might"
- Added on page 16, line 373-374: "(…) to detect potential toxic side effects of the treatment"
- Added on page 16, line 380: "(CRS)"
- Added in the figure legends the number of mice for each treatment group to each figure section to adapt closely to the journal's guidelines.
- Added more consistency in the manuscript: Used everywhere in the text and in the figures greek letters instead of latin letters, when appropriate (IFNγ instead of IFNg and TNFα instead of TNFa)

- Modified Figure 3
  - ➢ Included in subfigure 3A, column II two additional values in "Jinx+ATCT endogenous cells" (of now n = 15), that had been excluded by mistake (the mistake was realized during determining n of all individual values) → statistics did not change
  - ➢ so that the axis are better aligned with each other
- Moved Gating strategy (former Figure EV1A) to Figure EV5E
- Moved analysis of transferred cells (former Figure EV1B) to Figure EV2A and virus titres (former Figure EV2A) to Figure EV2B.
  - ➢ Modified text, figure legends etc. accordingly
- Modified Figure EV3C so that the axis titles were angled and therefore easier to distinguish the compared treatment groups. Modified legend for Figure EV3 to clarify that transferred T cells were compared with WT T cells.
- Added the following sentence to the acknowledgements: "The synopsis image for this publication was created with BioRender.com."

19th Sep 2022

Dear Dr. Aichele,

Thank you for the submission of your manuscript to EMBO Molecular Medicine. We have now received the enclosed reports from the two reviewers, and as you will see, they are supportive of publication. I am therefore pleased to inform you that we will be able to accept your manuscript once the following points will be addressed:

1/ Material and methods:
- Please provide the dilutions for the antibodies used in the study.
- Please indicate the gender of the mice used in the study.
- Human samples: Please include a statement that informed consent was obtained from all subjects (including healthy donors) and that the experiments conformed to the principles set out in the WMA Declaration of Helsinki and the Department of Health and Human Services Belmont Report. Please correct the checklist accordingly. If only samples that had been collected in a previous study were used, please mention it explicitly.

2/ Figures:
Please provide exact p values in all figures or in their legends (including for non-significant values, ns). Some people found that to keep the figures clear, providing a supplemental table with all exact p-values was preferable. You are welcome to do this if you want to.

3/ We would also strongly encourage you to include the source data for figure panels that show essential data. Numerical data should be provided as individual .xls or .csv files (including a tab describing the data). For blots or microscopy, uncropped images should be submitted (using a zip archive if multiple images need to be supplied for one panel). Additional information on source data and instruction on how to label the files are available at
.

4/ Thank you for providing a synopsis text and image. Please resize the visual abstract as a JPEG/TIFF/PNG file 550 px wide x 300-600 px high and make sure that the text remains legible. Please note that these would be the final versions and changes during proofing are usually not allowed.

5/ As part of the EMBO Publications transparent editorial process initiative (see our Editorial at http://embomolmed.embopress.org/content/2/9/329), EMBO Molecular Medicine will publish online a Review Process File (RPF) to accompany accepted manuscripts.
This file will be published in conjunction with your paper and will include the anonymous referee reports, your point-by-point response and all pertinent correspondence relating to the manuscript. Let us know whether you agree with the publication of the RPF and as here, if you want to remove or not any figures from it prior to publication.
Please note that the Authors checklist will be published at the end of the RPF.

I look forward to receiving your revised manuscript.

Yours sincerely,

Lise Roth

Lise Roth, PhD
Senior Editor
EMBO Molecular Medicine

.

***** Reviewer's comments *****

Referee #3 (Comments on Novelty/Model System for Author):

In the revised version, the authors provide convincing data that the phenotype of activated T cells in mouse models fairly closely resembles those of patients with untreated HLH. These would underscore the translational potential of their study and represent an important step towards developing gene-corrected autologous T cells for the treatment of HLH.

Referee #3 (Remarks for Author):

The authors have adequately addressed my previous concerns. In particular, more detailed comparison of human and mouse T cell phenotypes would indicate that the phenotype of activated T cells observed in the mouse models resembles those of patients with untreated HLH. The revision has further strengthened the manuscript. I have no further comments.

Referee #4 (Comments on Novelty/Model System for Author):

The authors have presented an interesting set of experiments in an animal model. While there are additional experiments that could be done to improve the impact, the authors argue these will be the subject of future work. I feel this reduces impact some, but I accept their point that there is already a great deal of work and some novelty in the paper as it stands and that the additional experiments are likely to take several months.

The authors performed the requested editorial changes.

29th Sep 2022

Dear Dr. Aichele,

Thank you for submitting your revised files. I am pleased to inform you that your manuscript is accepted for publication and is now being sent to our publisher to be included in the next available issue of EMBO Molecular Medicine.

Congratulations on your interesting work!

With kind regards,

Lise Roth

Lise Roth, Ph.D
Senior Editor
EMBO Molecular Medicine

EMBO Press Author Checklist

Corresponding Author Name: Dr. Peter Aichele	
Journal Submitted to: EMBO Molecular Medicine	
Manuscript Number: EMM-2022-16085	

USEFUL LINKS FOR COMPLETING THIS FORM
The EMBO Journal - Author Guidelines
EMBO Reports - Author Guidelines
Molecular Systems Biology - Author Guidelines
EMBO Molecular Medicine - Author Guidelines

Reporting Checklist for Life Science Articles (updated January 2022)
This checklist is adapted from Materials Design Analysis Reporting (MDAR) Checklist for Authors. MDAR establishes a minimum set of requirements in transparent reporting in the life sciences (see Statement of Task: 10.31222/osf.io/9sm4x). Please follow the journal's guidelines in preparing your manuscript.
Please note that a copy of this checklist will be published alongside your article.

Abridged guidelines for figures
1. Data
The data shown in figures should satisfy the following conditions:
- the data were obtained and processed according to the field's best practice and are presented to reflect the results of the experiments in an accurate and unbiased manner.
- ideally, figure panels should include only measurements that are directly comparable to each other and obtained with the same assay.
- plots include clearly labeled error bars for independent experiments and sample sizes. Unless justified, error bars should not be shown for technical replicates.
- if n<5, the individual data points from each experiment should be plotted. Any statistical test employed should be justified.
- Source Data should be included to report the data underlying figures according to the guidelines set out in the authorship guidelines on Data Presentation.

2. Captions
Each figure caption should contain the following information, for each panel where they are relevant:
- a specification of the experimental system investigated (eg cell line, species name).
- the assay(s) and method(s) used to carry out the reported observations and measurements.
- an explicit mention of the biological and chemical entity(ies) that are being measured.
- an explicit mention of the biological and chemical entity(ies) that are altered/varied/perturbed in a controlled manner.
- the exact sample size (n) for each experimental group/condition, given as a number, not a range;
- a description of the sample collection allowing the reader to understand whether the samples represent technical or biological replicates (including how many animals, litters, cultures, etc.).
- a statement of how many times the experiment shown was independently replicated in the laboratory.
- definitions of statistical methods and measures:
  - common tests, such as t-test (please specify whether paired vs. unpaired), simple χ2 tests, Wilcoxon and Mann-Whitney tests, can be unambiguously identified by name only, but more complex techniques should be described in the methods section;
  - are tests one-sided or two-sided?
  - are there adjustments for multiple comparisons?
  - exact statistical test results, e.g., P values = x but not P values < x;
  - definition of 'center values' as median or average;
  - definition of error bars as s.d. or s.e.m.

Please complete ALL of the questions below.
Select "Not Applicable" only when the requested information is not relevant for your study.

Materials

Newly Created Materials	Information included in the manuscript?	In which section is the information available? (Reagents and Tools Table, Materials and Methods, Figures, Data Availability Section)
New materials and reagents need to be available; do any restrictions apply?	Not Applicable	

Antibodies	Information included in the manuscript?	In which section is the information available? (Reagents and Tools Table, Materials and Methods, Figures, Data Availability Section)
For **antibodies** provide the following information:<br>- Commercial antibodies: RRID (if possible) or supplier name, catalogue number and or/clone number<br>- Non-commercial: RRID or citation	Yes	Materials and Methods

DNA and RNA sequences	Information included in the manuscript?	In which section is the information available? (Reagents and Tools Table, Materials and Methods, Figures, Data Availability Section)
**Short novel DNA or RNA including primers, probes**: provide the sequences.	Not Applicable	

Cell materials	Information included in the manuscript?	In which section is the information available? (Reagents and Tools Table, Materials and Methods, Figures, Data Availability Section)
**Cell lines:** Provide species information, strain. Provide accession number in repository **OR** supplier name, catalog number, clone number, and/**OR** RRID.	Not Applicable	
**Primary cultures:** Provide species, strain, sex of origin, genetic modification status.	Not Applicable	
Report if the cell lines were recently **authenticated** (e.g., by STR profiling) and tested for mycoplasma contamination.	Not Applicable	

Experimental animals	Information included in the manuscript?	In which section is the information available? (Reagents and Tools Table, Materials and Methods, Figures, Data Availability Section)
**Laboratory animals or Model organisms:** Provide species, strain, sex, age, genetic modification status. Provide accession number in repository **OR** supplier name, catalog number, clone number, **OR** RRID.	Yes	Materials and Methods
**Animal observed in or captured from the field:** Provide species, sex, and age where possible.	Not Applicable	
Please detail **housing and husbandry conditions**.	Yes	Materials and Methods

Plants and microbes	Information included in the manuscript?	In which section is the information available? (Reagents and Tools Table, Materials and Methods, Figures, Data Availability Section)
**Plants:** provide species and strain, ecotype and cultivar where relevant, unique accession number if available, and source (including location for collected wild specimens).	Not Applicable	
**Microbes:** provide species and strain, unique accession number if available, and source.	Yes	Materials and Methods

Human research participants	Information included in the manuscript?	In which section is the information available? (Reagents and Tools Table, Materials and Methods, Figures, Data Availability Section)
If collected and within the bounds of privacy constraints report on age, sex and gender or ethnicity for all study participants.	Not Applicable	

Core facilities	Information included in the manuscript?	In which section is the information available? (Reagents and Tools Table, Materials and Methods, Figures, Data Availability Section)
If your work benefited from core facilities, was their service mentioned in the acknowledgments section?	Not Applicable	

Design

Study protocol	Information included in the manuscript?	In which section is the information available? (Reagents and Tools Table, Materials and Methods, Figures, Data Availability Section)
If study protocol has been **pre-registered, provide DOI in the manuscript**. For clinical trials, provide the trial registration number **OR** cite DOI.	Yes	Materials and Methods
Report the **clinical trial registration number** (at ClinicalTrials.gov or equivalent), where applicable.	Not Applicable	

Laboratory protocol	Information included in the manuscript?	In which section is the information available? (Reagents and Tools Table, Materials and Methods, Figures, Data Availability Section)
Provide DOI OR other citation details if **external detailed step-by-step protocols** are available.	Not Applicable	

Experimental study design and statistics	Information included in the manuscript?	In which section is the information available? (Reagents and Tools Table, Materials and Methods, Figures, Data Availability Section)
Include a statement about **sample size** estimate even if no statistical methods were used.	Yes	Materials and Methods, Figure legends
Were any steps taken to minimize the effects of subjective bias when allocating animals/samples to treatment (e.g. **randomization procedure**)? If yes, have they been described?	Not Applicable	
Include a statement about **blinding** even if no blinding was done.	Not Applicable	
Describe **inclusion/exclusion criteria** if samples or animals were excluded from the analysis. Were the criteria pre-established?<br><br>If sample or data points were omitted from analysis, report if this was due to attrition or intentional exclusion and provide justification.	Not Applicable	
For every figure, are **statistical tests** justified as appropriate? Do the data meet the assumptions of the tests (e.g., normal distribution)? Describe any methods used to assess it. Is there an estimate of variation within each group of data? Is the variance similar between the groups that are being statistically compared?	Yes	Materials and Methods, Figure legends

Sample definition and in-laboratory replication	Information included in the manuscript?	In which section is the information available? (Reagents and Tools Table, Materials and Methods, Figures, Data Availability Section)
In the figure legends: state number of times the experiment was **replicated** in laboratory.	Yes	Figure legends
In the figure legends: define whether data describe **technical or biological replicates**.	Yes	Figure legends

Ethics

Ethics	Information included in the manuscript?	In which section is the information available? (Reagents and Tools Table, Materials and Methods, Figures, Data Availability Section)
Studies involving **human participants**: State details of **authority granting ethics approval** (IRB or equivalent committee(s), provide reference number for approval.	Yes	Materials and Methods
Studies involving **human participants**: Include a statement confirming that **informed consent** was obtained from all subjects and that the experiments conformed to the principles set out in the WMA Declaration of Helsinki and the Department of Health and Human Services Belmont Report.	Yes	Materials and Methods
Studies involving **human participants:** For publication of **patient photos**, include a statement confirming that consent to publish was obtained.	Not Applicable	
Studies involving experimental **animals**: State details of **authority granting ethics approval** (IRB or equivalent committee(s), provide reference number for approval. Include a statement of compliance with ethical regulations.	Yes	Mouse experiments were approved by Regierungspräsidium Freiburg (G-16/06, G-17/99, G-18/125).
Studies involving **specimen and field samples:** State if relevant **permits** obtained, provide details of authority approving study; if none were required, explain why.	Not Applicable	

Dual Use Research of Concern (DURC)	Information included in the manuscript?	In which section is the information available? (Reagents and Tools Table, Materials and Methods, Figures, Data Availability Section)
Could your study fall under dual use research restrictions? Please check biosecurity documents and list of **select agents and toxins** (CDC): https://www.selectagents.gov/sat/list.htm	Not Applicable	
If you used a select agent, is the security level of the lab appropriate and reported in the manuscript?	Not Applicable	
If a study is subject to dual use research of concern regulations, is the name of the **authority granting approval and reference number** for the regulatory approval provided in the manuscript?	Not Applicable	

Reporting

The MDAR framework recommends adoption of discipline-specific guidelines, established and endorsed through community initiatives. Journals have their own policy about requiring specific guidelines and recommendations to complement MDAR.

Adherence to community standards	Information included in the manuscript?	In which section is the information available? (Reagents and Tools Table, Materials and Methods, Figures, Data Availability Section)
State if relevant guidelines or checklists (e.g., **ICMJE, MIBBI, ARRIVE, PRISMA**) have been followed or provided.	Yes	Mouse experiments were conducted according to ARRIVE guidelines.
For **tumor marker prognostic studies,** we recommend that you follow the **REMARK** reporting guidelines (see link list at top right). See author guidelines, under 'Reporting Guidelines'. Please confirm you have followed these guidelines.	Not Applicable	
For **phase II and III randomized controlled trials**, please refer to the **CONSORT** flow diagram (see link list at top right) and submit the CONSORT checklist (see link list at top right) with your submission. See author guidelines, under 'Reporting Guidelines'. Please confirm you have submitted this list.	Not Applicable	

Data Availability

Data availability	Information included in the manuscript?	In which section is the information available? (Reagents and Tools Table, Materials and Methods, Figures, Data Availability Section)
Have **primary datasets** been deposited according to the journal's guidelines (see 'Data Deposition' section) and the respective accession numbers provided in the Data Availability Section?	Not Applicable	
Were **human clinical and genomic datasets** deposited in a public access-controlled repository in accordance to ethical obligations to the patients and to the applicable consent agreement?	Not Applicable	
Are **computational models** that are central and integral to a study available without restrictions in a machine-readable form? Were the relevant accession numbers or links provided?	Not Applicable	
If publicly available data were reused, provide the respective **data citations in the reference list**.	Not Applicable	
